# Plant responses to heat stress and advances in mitigation strategies

**DOI:** 10.3389/fpls.2025.1638213

**Published:** 2025-08-29

**Authors:** Abay T. Samat, Aigerim Soltabayeva, Assemgul Bekturova, Kuralay Zhanassova, Dana Auganova, Zhaksylyk Masalimov, Sudhakar Srivastava, Mereke Satkanov, Assylay Kurmanbayeva

**Affiliations:** ^1^ Department of Biotechnology and Microbiology, L.N. Gumilyov Eurasian National University, Astana, Kazakhstan; ^2^ LLP “Biosense”, Ust-Kamenogorsk, Kazakhstan; ^3^ Biology Department, School of Science and Humanities, Nazarbayev University, Astana, Kazakhstan; ^4^ National Certification System for Tissue Culture Raised Plants, National Institute of Plant Genome Research, New Delhi, India

**Keywords:** abiotic stress, high temperature, adaptive changes, morphological parameters, physiological parameters, mitigation strategies

## Abstract

High-temperature stress is a major abiotic constraint limiting plant growth and agricultural productivity. While its adverse effects are well documented, most studies have examined individual species or isolated physiological mechanisms. This review provides a comprehensive comparative analysis of heat stress responses across four major crops - barley (*Hordeum vulgare*), rice (*Oryza sativa*), maize (*Zea mays*), and tomato (*Solanum lycopersicum*), alongside the model plant *Arabidopsis thaliana*, focusing on their morphological, physiological, and biochemical adaptations as well as current mitigation strategies. Morphological assessments reveal that root traits are more heat-sensitive than shoot length, biomass, or germination rate. Physiologically, all species exhibit reduced photosynthetic rate and PSII efficiency (Fv/Fm), though stomatal conductance and transpiration responses vary. Biochemically, the accumulation of reactive oxygen species (ROS) and antioxidant activity exhibit species- and stress-dependent regulation, with both upregulation and downregulation observed. Among mitigation approaches, seed priming emerges as a cost-effective strategy, while miRNA-mediated regulation shows strong potential for developing heat-tolerant cultivars. This synthesis highlights critical knowledge gaps and outlines future directions for enhancing crop resilience in the face of rising temperatures.

## 1 Introduction

Recent urbanization and population growth have placed unprecedented pressure on agricultural systems, while climate change has emerged as one of the critical threats to global crop productivity (Kousar et al., 2021; Benitez-Alfonso et al., 2023; Farooq et al., 2023; Terán et al., 2024). The accelerating pace of global warming is particularly alarming, with 2024 projected to be the warmest year on record, approximately 1.55°C above pre-industrial levels (WMO, 2024). Climate models suggest 66% probability that at least one year between 2024 and 2028 will temporarily exceed the 1.5°C threshold, with sustained exceedance likely by the early 2030s (IPCC, 2023). This warming trend poses severe challenges to agriculture, where heat stress, exacerbated by the increasing frequency of extreme weather events, significantly compromises crop yields and quality (Mirón et al., 2023; Janni et al., 2024). Meta-analyses indicate that each 1°C increase in temperature reduces the yield of major crops by 3–7% (Challinor et al., 2014; Zhao et al., 2017; Tran et al., 2025), creating substantial risks to global food security as demand is projected to rise by 50% by 2050 (FAO, 2021). Addressing these challenges requires both innovative mitigation strategies and a deeper understanding of plant responses to heat stress to facilitate breeding of climate-resilient varieties (Kousar et al., 2021; Terán et al., 2024).

Although plant responses to heat stress have been extensively studied, most reviews focus on individual species or specific mechanisms, limiting broad interspecific comparisons (Wu et al., 2019; Jacott and Boden, 2020; Medina et al., 2021; Elakhdar et al., 2022; El-Sappah et al., 2022; LI et al., 2022; Singh et al., 2022; Ren et al., 2023; Sarma et al., 2023; Ruan et al., 2024). Similarly, comprehensive studies on *Oryza sativa* (rice) emphasize varietal differences in thermotolerance, hormonal regulation, and physiological adaptations (Wu et al., 2019; Ren et al., 2023; Sarma et al., 2023). In *Zea mays* (maize), heat stress responses have been dissected from molecular, transcriptional, and agronomic perspectives, while studies on *Solanum lycopersicum* (Tomato) focus on reactive oxygen species (ROS) signaling and biochemical adjustments (Medina et al., 2021; El-Sappah et al., 2022; LI et al., 2022; Singh et al., 2022; Ruan et al., 2024). Despite these advances, a systematic comparison across species is lacking. Our review addresses this critical knowledge gap through a comprehensive interspecific analysis of five representative species – *Hordeum vulgare*, *Oryza sativa*, *Zea mays*, *Solanum lycopersicum*, and *Arabidopsis thaliana* – spanning both monocots and dicots. These species were selected based on their agronomic importance, diverse growth habits, photosynthetic pathways, ecological adaptations, and availability of heat stress response data, with *A. thaliana* included as a well-established model plant. We systematically evaluate their morphological, physiological, and biochemical adaptation to heat stress across different experimental systems.

Beyond assessing stress responses, this review critically compares current mitigation strategies, which are often examined in isolation rather than across multiple species (Liu et al., 2022; Zhang et al., 2022; Feng et al., 2023; Iqbal et al., 2023; Louis et al., 2023; Ma and Hu, 2023). Seed priming, for example, enhances thermotolerance by boosting antioxidant activity, inducing stress memory, and even conferring transgenerational resilience (Liu et al., 2022; Louis et al., 2023). Chemical and nutritional treatments mitigate heat stress through osmotic regulation and antioxidant defense activation, while microbial biostimulants improve tolerance via beneficial plant–microbe interactions (Feng et al., 2023; Iqbal et al., 2023). Additionally, miRNAs have gained attention as key post-transcriptional regulators of heat stress signaling and adaptation (Zhang et al., 2022; Ma and Hu, 2023). However, most of these studies evaluate these strategies in a species-specific context, obscuring broader trends. Here, we systematically analyze four major mitigation approaches across five species, assessing comparative efficacy and providing a unified perspective on heat stress management in crops.

## 2 Morphological changes in plants under heat stress

Each plant species possesses unique optimal temperature conditions, indispensable for sustaining normal growth and development (Krutovsky et al., 2025). These conditions can exhibit considerable variation depending on the species’ genetic background and ecological origins. When the temperatures exceed this range, morphological disruptions occur, including reduced seed germination, abnormal root and shoot development, and overall growth impairment. These changes serve as critical indicators of heat stress damage, particularly in economically important crops such as *H. vulgare*, *O. sativa*, *Z. mays*, *S. lycopersicum*, and the model plant *Arabidopsis thaliana* (Ito et al., 2009; Hussain et al., 2019a; Naz et al., 2022).

### 2.1 Seed germination response to high temperatures

Seed germination is one of the most heat-sensitive stages of plant development, making it a key marker for assessing heat stress effects (Scandalios et al., 2000; Mei and Song, 2010; Liu et al., 2015). Supra-optimal temperatures disrupt germination, essential for successful plant establishment and growth (**Table 1**) (Baskin and Baskin, 2000; Brändel, 2004). Above 30°C, germination rates decline sharply, and under extreme heat (>45°C), germination may be severely reduced or completely inhibited (Liu et al., 2015).

**Table 1:** Germination rate reduction in different plant species under heat stress.

Plant species	Decrease in germination	T°C	Duration of heat stress treatment	References
*Hordeum vulgare*	95.3% ↓	40°C	8 days	Mei and Song, 2010
*Oryza sativa*	90% ↓	45°C	7 days	Liu et al., 2015
*Zea mays*	40% ↓	40°C	10 days	Scandalios et al., 2000
*Solanum lycopersicum*	50% ↓	40°C	8 days	Tokić et al., 2023
*Arabidopsis thaliana*	75% ↓	30°C	8 days	Afshar et al., 2016

Arrows heads down (↓) represent negative changes. T°C - temperature in Celsius.

As shown in **Table 1**, the extent of germination reduction varies among plant species. For example, germination rates decrease by 95.3% in barley cv. Pijiu, 90% in rice cv. Peiai, 50% in tomato cv. C38, and 75% in *Arabidopsis* cv. Columbia (Labouriau and Osborn, 1984; Mei and Song, 2010; Liu et al., 2015; Afshar et al., 2016; Tokić et al., 2023). In contrast, heat-tolerant maize cultivars, w64a, r6-67, and dn-6, exhibit a more moderate decline (~40%), highlighting genotypic variation in thermotolerance (Scandalios et al., 2000).

However, laboratory-based germination studies may not fully reflect field conditions, where interacting environmental factors (temperature fluctuations, humidity, light, soil nutrients) influence seed behavior (Klupczyńska and Pawłowski, 2021; Qaderi, 2023). While controlled experiments provide valuable insights into specific physiological mechanisms, they often fail to capture ecological complexity and may overestimate seed germination resilience. Field conditions present a more challenging environment where seeds encounter unpredictable stress combinations – including diurnal temperature fluctuations, variable soil moisture, and biotic interactions – that collectively alter germination outcomes compared to uniform laboratory conditions. Additionally, germination is only significantly affected under extreme heat, showing low sensitivity at moderate stress levels. Thus, relying solely on germination data may be insufficient for predicting overall plant productivity under heat stress, and it requires a broader focus, incorporating subsequent morphological parameters.

### 2.2 Impact of heat stress on root and shoot growth

Elevated temperatures severely suppress root and shoot expansion, leading to reduced biomass accumulation. Impaired root function under heat stress disrupts water and nutrient uptake, further exacerbating growth limitations (Koevoets et al., 2016; Taratima et al., 2022). Among the analyzed species, root fresh weight (RFW) emerges as one of the most heat-sensitive root parameters (**Table 2**). *Arabidopsis*, rice, and barley show severe reductions in RFW, ranging from 52% to 70% under thermal stress, while maize and tomato exhibit more moderate declines of 29% and 39%, respectively (Abo Gamar et al., 2019; Hussain et al., 2019a; Guo et al., 2022; Naz et al., 2022; Baloch et al., 2024). This pattern extends to reduce root dry weight (RDW), where similar percentage reductions suggest parallel impacts on both biomass accumulation and water retention capacity (Xia et al., 2021; Guo et al., 2022; Macabuhay et al., 2022; Naz et al., 2022; Baloch et al., 2024).

**Table 2:** Heat stress-induced reduction in root fresh and dry weight.

Plant species	Decrease in root weight	T°C	Duration of heat stress treatment	References
Root fresh weight				
*Hordeum vulgare*	52% ↓	34°C	12 days	Naz et al., 2022
*Oryza sativa*	60% ↓	40°C	7 days	Baloch et al., 2024
*Zea mays*	29% ↓	35-40°C	15 days	Hussain et al., 2019a
*Solanum lycopersicum*	39% ↓	40°C	4 weeks	Guo et al., 2022
*Arabidopsis thaliana*	~70% ↓	28°C	6 days	Abo Gamar et al., 2019
Root dry weight				
*Hordeum vulgare*	30% ↓	34°C	12 days	Naz et al., 2022
*Oryza sativa*	59% ↓	40°C	7 days	Baloch et al., 2024
*Zea mays*	29.17% ↓	36°C	8 days	Xia et al., 2021
*Solanum lycopersicum*	50.8% ↓	40°C	4 weeks	Guo et al., 2022
*Arabidopsis thaliana*	50% ↓	30°C	20 days	Macabuhay et al., 2022

Arrows heads down (↓) represent negative changes. ~ indicates approximately. T°C, temperature in Celsius.

While both root and shoot biomass are temperature-sensitive, shoot fresh weight (SFW) generally displays greater stress resilience than root parameters (**Table 3**). Significant SFW reductions (29-50%) occur in barley, tomato, Arabidopsis, and rice, contrasting with maize’s notably higher tolerance (only 3.2-8% reduction) (Hussain et al., 2016; Abo Gamar et al., 2019; Zhou et al., 2019; Naz et al., 2022; Baloch et al., 2024). This suggests that despite the negative effects of heat stress, some plant species exhibit a higher capacity to maintain shoot and leaf biomass production. This interspecific variation in shoot response is further evidenced by shoot dry weight (SDW) measurements, where *Arabidopsis* and barley suffer substantial losses (20-25%), compared to more modest impacts on maize (3%), tomato (8%), and rice (11%), highlighting fundamental differences in thermotolerance mechanisms among species (Ito et al., 2009; Hussain et al., 2016; Zhou et al., 2019; Macabuhay et al., 2022; Naz et al., 2022).

**Table 3:** Heat stress-induced reduction in shoot fresh and dry weight.

Plant species	Decrease in shoot weight	T°C	Duration of heat stress treatment	References
Shoot fresh weight				
*Hordeum vulgare*	29% ↓	34°C	12 days	Naz et al., 2022
*Oryza sativa*	50% ↓	40°C	7 days	Baloch et al., 2024
*Zea mays*	3.2% - 8% ↓	38°C	15 days	Hussain et al., 2016
*Solanum lycopersicum*	33% ↓	26°C	6 days	Zhou et al., 2019
*Arabidopsis thaliana*	~50% ↓	28°C	6 days	Abo Gamar et al., 2019
Shoot dry weight				
*Hordeum vulgare*	25% ↓	34°C	12 days	Naz et al., 2022
*Oryza sativa*	11% ↓	35°C	7 days	Ito et al., 2009
*Zea mays*	3.35% - 3.75% ↓	38°C	15 days	Hussain et al., 2016
*Solanum lycopersicum*	8% ↓	26°C	6 days	Zhou et al., 2019
*Arabidopsis thaliana*	20% ↓	30°C	20 days	Macabuhay et al., 2022

Arrows heads down (↓) represent negative changes. ~ indicates approximately. T°C, temperature in Celsius.

In addition to reducing biomass, heat stress profoundly influences root and shoot elongation (**Table 4**). The extent of these reductions varies among species, reflecting differences in their ability to tolerate high temperatures. Root length reductions vary from 12% in rice cv. Vandana, 26% in barley, 48% in tomato, to 55% in *Arabidopsis* cv. Columbia (Sailaja et al., 2014; Yang et al., 2017; Guo et al., 2022; Naz et al., 2022). A similar trend is observed for shoot length, with reductions of 5%-10% in Arabidopsis, 14% in barley and rice, and 59.1% in tomato under heat-stress conditions (Sailaja et al., 2014; Ibañez et al., 2017; Guo et al., 2022; Naz et al., 2022). Maize cv. SD609 again demonstrates exceptional thermal stability, with only 35% root length reduction and minimal shoot length impact (6-8%), reinforcing its status as a heat-resilient crop (Hussain et al., 2016; Xia et al., 2021). This relative stability in shoot growth suggests species-specific adaptations to thermal stress.

**Table 4:** Heat stress-induced reduction in root and shoot length.

Plant species	Decrease in length	T°C	Duration of heat stress treatment	References
Root length				
*Hordeum vulgare*	26% ↓	34°C	12 days	Naz et al., 2022
*Oryza sativa*	12% ↓	42°C	5 days	Sailaja et al., 2014
*Zea mays*	35.42% ↓	36°C	8 days	Xia et al., 2021
*Solanum lycopersicum*	48.07% ↓	40°C	4 weeks	Guo et al., 2022
*Arabidopsis thaliana*	55% ↓	30°C	10 days	Yang et al., 2017
Shoot length				
*Hordeum vulgare*	14% ↓	34°C	12 days	Naz et al., 2022
*Oryza sativa*	14% ↓	42°C	5 days	Sailaja et al., 2014
*Zea mays*	6.4% - 8.8% ↓	38°C	15 days	Hussain et al., 2016
*Solanum lycopersicum*	59.1% ↓	40°C	4 weeks	Guo et al., 2022
*Arabidopsis thaliana*	5 - 10% ↓	28°C	10 days	Ibañez et al., 2017

Arrows heads down (↓) represent negative changes. T°C, temperature in Celsius.

**Figure 1:**
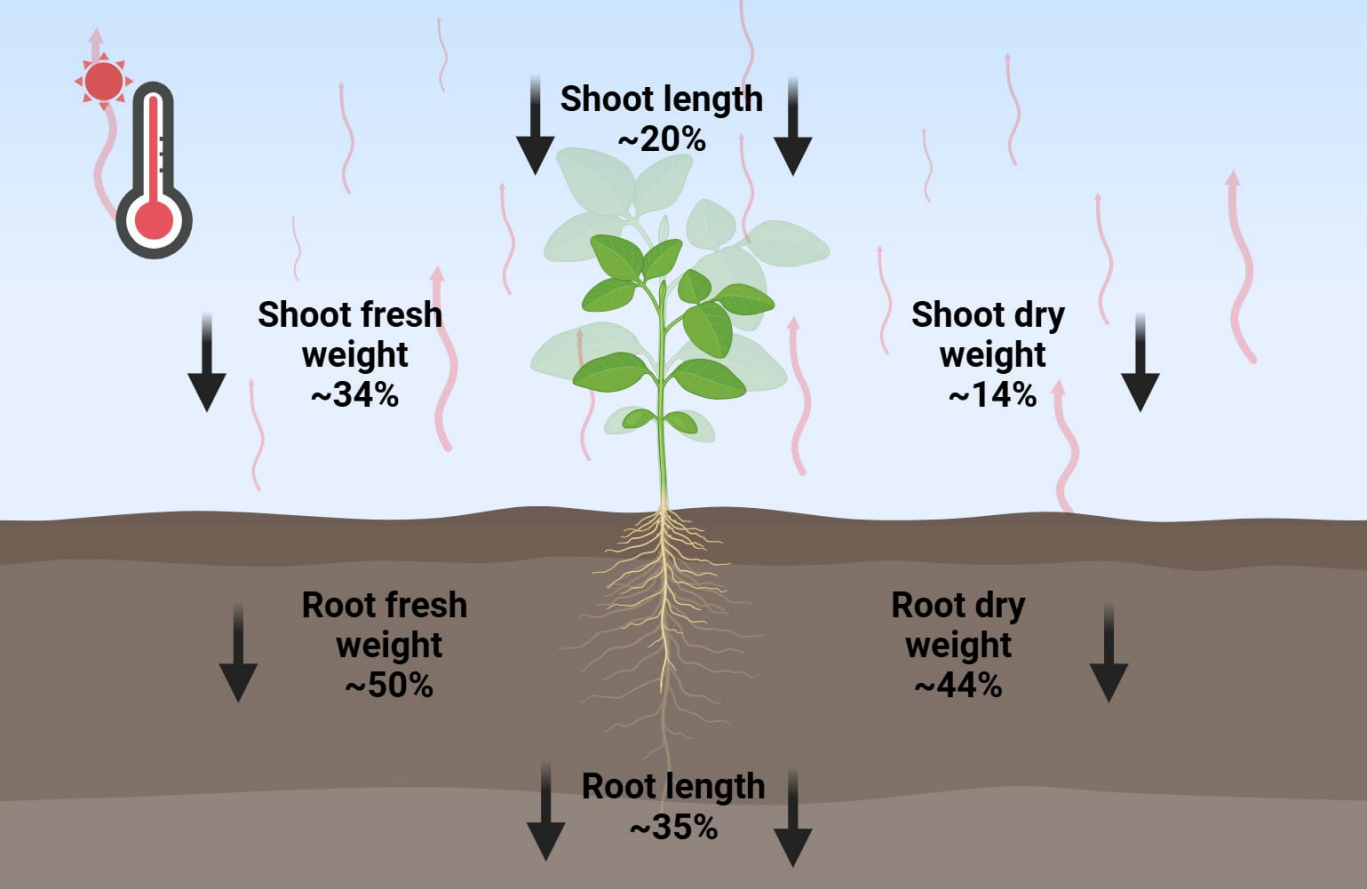
Effect of heat stress on root and shoot growth parameters. This figure presents species-averaged data illustrating the impact of heat stress on various crop species, as detailed in **Tables 2**-**4**. Red wavy lines indicate an increase in temperature; Bold black arrows represent the reduction of growth parameters.

These morphological alterations directly impair critical physiological functions, including nutrient uptake, water balance, and overall metabolic homeostasis, collectively compromising normal growth and development (Yamaura et al., 2021; Barten et al., 2022; Pérez-Bueno et al., 2022; Visakorpi et al., 2023). The synergistic effects of diminished biomass production, restricted elongation growth, and impaired hydration status ultimately lead to severe yield penalties, particularly in heat-sensitive species (**Figure 1**). At the cellular level, these macroscopic changes reflect underlying disruptions in hormone signaling, oxidative homeostasis, and carbon metabolism, with suppressed photosynthetic capacity (manifested through reduced leaf expansion, limited biomass production, and stunted growth) representing a key factor exacerbating heat stress impacts on plant performance and productivity (Greer and Weedon, 2012; Brunel-Muguet et al., 2015; Mahmood et al., 2021, 2022; Bernacchi et al., 2023; Burroughs et al., 2023; Batool et al., 2025; Li et al., 2025; Secomandi et al., 2025).

## 3 Formatting physiological changes in plants under heat stress

High temperatures significantly impair critical physiological processes, particularly photosynthetic efficiency, which serves as a key indicator of photosystem functionality. Essential parameters affected include the maximum quantum efficiency of photosystem II (PSII), stomatal conductance, and transpiration rate - all crucial for proper plant growth and development (Rollins et al., 2013; Zheng et al., 2025). These head-induced disruptions lead to diminished carbon assimilation, reduced water use efficiency, and overall growth inhibition.

### 3.1 Photosynthetic parameters

One of the primary consequences of heat stress is the inactivation of Rubisco, accompanied by reductions in chlorophyll content and PSII efficiency, collectively causing a substantial decline in photosynthetic activity (Wijewardene et al., 2021; Qu et al., 2023; Scafaro et al., 2023; Zahra et al., 2023). Experimental data (**Table 5**) demonstrate consistent reductions in both photosynthesis rate and PSII maximum quantum yield (Fv/Fm) across species: photosynthesis decreases by 30-50*%* in barley, 23% in rice, 16.6% in maize, 20% in tomato, and 16% in *Arabidopsis* (Vile et al., 2012; Zhou et al., 2017; Hussain et al., 2019a; Yang et al., 2020; Mahalingam et al., 2022). Similarly, Fv/Fm values decrease under stress, with reductions of 66.6% in rice, 20% in *Arabidopsis*, 7.5% in barley, 4% in maize, and 7% in tomato (Rollins et al., 2013; Halter et al., 2017; Zhou et al., 2017; Yang et al., 2018; Zhang et al., 2018; Doğru, 2021). These species-specific response patterns highlight distinct thermotolerance mechanisms among plants, with maize exhibiting relatively greater photosynthetic stability under heat stress compared to more sensitive species like rice and barley.

**Table 5:** Negative effects of heat stress on photosynthetic activity.

Plant species	Decrease in photosynthetic activity %	T°C	Duration of heat stress treatment	References
Photosynthesis rate				
*Hordeum vulgare*	~30-50% ↓	36°C	6 days	Mahalingam et al., 2022
*Oryza sativa*	~23% ↓	38°C	9 days	Yang et al., 2020
*Zea mays*	~16.6% ↓	38°C	15 days	Hussain et al., 2016
*Solanum lycopersicum*	20% ↓	36°C	4 days	Zhou et al., 2017
*Arabidopsis thaliana*	16% ↓	30°C	20 days	Vile et al., 2012
Maximum quantum yield of photosystem II (Fv/Fm)				
*Hordeum vulgare*	~7.5% ↓	36°C	7 days	Rollins et al., 2013
*Oryza sativa*	~66.6% ↓	43°C	1–3 hours	Yang et al., 2018; Zhang et al., 2018
*Zea mays*	~4% ↓	45°C	20 mins	Doğru, 2021
*Solanum lycopersicum*	7.1% ↓	36°C	4 days	Zhou et al., 2017
*Arabidopsis thaliana*	20% ↓	42°C	2–4 hours	Halter et al., 2017

Arrows heads down (↓) represent negative changes. ~ indicates approximately. T°C, temperature in Celsius.

The detrimental effects of heat stress extend beyond simple reductions in chlorophyll content and PSII efficiency, significantly disrupting critical regulatory mechanisms including stomatal conductance and transpiration rate (Marchin et al., 2023; Zahra et al., 2023; Falcioni et al., 2024; Miao et al., 2025). Under elevated temperature, stomatal conductance – the crucial regulator of gas exchange and water loss – demonstrates a biphasic response: an initial increase to promote transpirational cooling, followed by a decline during prolonged heat exposure (Faralli et al., 2022; Marchin et al., 2022, 2023; Liang et al., 2023). This dynamic pattern exhibits considerable interspecies variation, reflecting distinct evolutionary adaptations to thermal stress. The intricate relationship between stomatal behavior, transpiration efficiency, and photosynthetic performance reveals the sophisticated nature of plant thermoregulation, where maintaining an optimal balance between water conservation and carbon fixation becomes paramount for survival under heat stress conditions.

### 3.2 Stomatal and transpiration responses to heat stress

Among the key physiological responses to heat stress, stomatal conductance is crucial in regulating gas exchange and transpiration. Studies show this parameter exhibits the most pronounced increase under elevated temperatures, with barley cultivars demonstrating a 50–80% rise after 5–15 days of exposure to 28°C and 38°C (Mahalingam et al., 2022). Similarly, other species also demonstrate an increase to varying degrees: rice (20%), *Arabidopsis* (11%), maize (10–30%), and decrease tomato (33%) (**Table 6**) (Jin et al., 2011; Hussain et al., 2016; Zhou et al., 2017; Yang et al., 2020; Mahalingam et al., 2022). These disparities highlight the importance of considering experimental context when interpreting heat stress responses, particularly regarding tissue specificity (leaves vs. whole seedlings), plant developmental stage, duration and intensity of heat exposure, and species-specific tolerance.

**Table 6:** Effects of heat stress on stomatal conductance and transpiration rate.

Plant species	Changes in %	T°C	Duration of heat stress treatment	References
Stomatal conductance				
*Hordeum vulgare*	~50-80% ↑	36°C	5 days	Mahalingam et al., 2022
*Oryza sativa*	~20% ↑	38°C	9 days	Yang et al., 2020
*Zea mays*	~10%-30% ↑	38°C	15 days	Hussain et al., 2016
*Solanum lycopersicum*	~33.3% ↓	36°C	4 days	Zhou et al., 2017
*Arabidopsis thaliana*	11% ↑	28°C	3 days	Jin et al., 2011
Transpiration rate				
*Hordeum vulgare*	~30% ↑	36°C	5 days	Mahalingam et al., 2022
*Oryza sativa*	~35-62% ↑	38°C	9 days	Yang et al., 2020
*Zea mays*	~9%- 6% ↑	38°C	15 days	Hussain et al., 2016
*Solanum lycopersicum*	~10% ↑	45°С	4 days	Zhou et al., 2017
*Arabidopsis thaliana*	12% ↑	28°C	3 days	Jin et al., 2011

Arrows’ heads up (↑) represent positive changes, and down (↓) represent negative changes. ~ indicates approximately. T°C, temperature in Celsius.

While short-term heat exposure typically enhances stomatal conductance, prolonged stress often leads to their decline, as observed in multiple tree species, including various oaks (*Quercus macrocarpa* Michx, *Q. muehlenbergii* Engl., *Q. stellata*), sweetgum (*Liquidambar styraciflua*), and other species like *Vitis vinifera L.*, *Ficus insipida* Willd., and *Ochroma pyramidale* (Hamerlynck and Knapp, 1996; Gunderson et al., 2002; Slot et al., 2016; Zhang et al., 2023; Veloo et al., 2025). Plants employ contrasting strategies to cope with heat stress - some temporarily increase stomatal opening for evaporative cooling while others rapidly close stomata to conserve water (Huang et al., 2025; Luo et al., 2025; Wu et al., 2025). These adaptive responses significantly influence water-use efficiency, photosynthetic performance, and long-term stress resilience.

Plants activate multiple mechanisms to counter prolonged heat stress, including transpirational cooling and stomatal regulation, which help balance water loss and cellular homeostasis (Mathur et al., 2014). However, while stomatal closure prevents dehydration, it simultaneously limits CO_2_ uptake, reducing photosynthetic efficiency and carbon assimilation (Hasanuzzaman et al., 2013; Faralli et al., 2022). Species-specific transpiration patterns reflect this balance, with the increase of 35-62% in rice, 30% in barley, 12% in *Arabidopsis*, 6–9% in maize, and 7% in tomato (**Table 6**). As highlighted by Jagadish et al., elevated transpiration coupled with effective leaf cooling can significantly mitigate heat stress impacts on plant growth and development (Jagadish et al., 2015). This underscores the critical need to optimize stomatal dynamics in crop breeding programs to enhance heat tolerance and maintain agricultural productivity under increasingly frequent and intense heat stress conditions.

**Figure 2:**
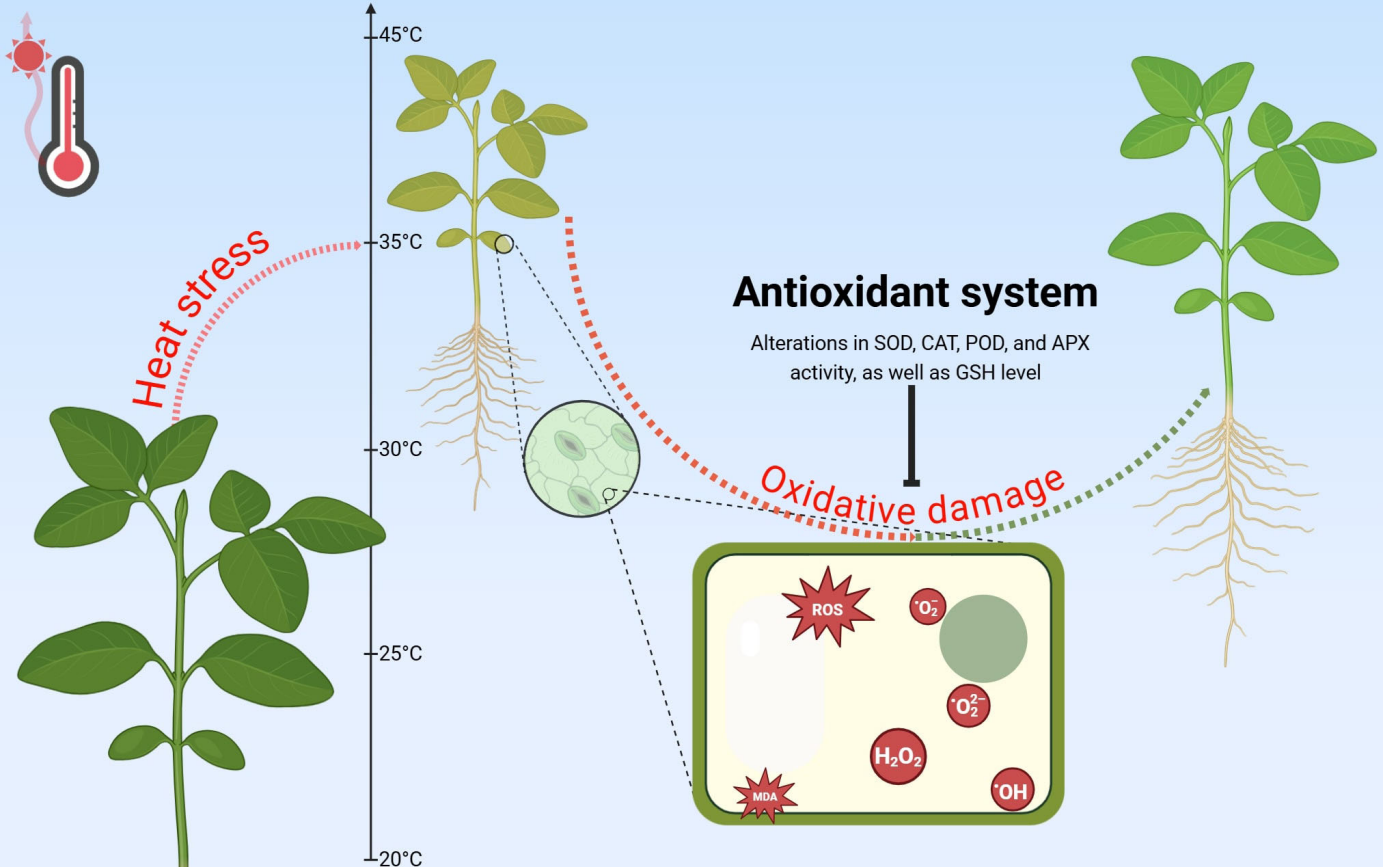
Physiological responses to heat stress. Red wavy lines indicate an increase in temperature; Bold black arrows represent the alterations of parameters.

While transpiration provides temporary heat relief, prolonged exposure disrupts fundamental photosynthetic processes (**Figure 2**). Elevated temperatures trigger complex responses, including altered reactive oxygen species (ROS) dynamics, reduced PSII (Fv/Fm) efficiency, and suppressed photosynthetic activity, collectively limiting carbon assimilation (Batool et al., 2025; Li et al., 2025; Ostendarp et al., 2025). The resulting decline in photosynthetic efficiency directly constrains plant growth and productivity. A comprehensive understanding of the interplay between stomatal behavior, transpiration, and photosynthesis is therefore essential for developing climate-resilient crops in our warming world (Zeng et al., 2017).

## 4 Biochemical changes in plants under heat stress

Environmental stressors such as extreme temperatures, drought, and salinity frequently induce oxidative stress in plants, leading to cellular damage (Gill and Tuteja, 2010; Li et al., 2023; Nurbekova et al., 2024; Rao and Zheng, 2025; Secomandi et al., 2025). Among these, heat stress poses a particularly severe threat to plant physiology by triggering excessive accumulation of reactive oxygen species (ROS) (Fortunato et al., 2023; Rao and Zheng, 2025). These highly reactive molecules, including hydrogen peroxide (H_2_O_2_) and superoxide (O_2_^-^), disrupt cellular processes and contribute to oxidative stress, resulting in membrane damage, impaired metabolism, and overall decline in plant health (Gill and Tuteja, 2010; Samat et al., 2024; Jardim-Messeder et al., 2025; Kračun et al., 2025).

### 4.1 Reactive oxygen species accumulation under heat stress

Heat stress induces substantial increases in ROS levels across various plant species, contributing to cellular damage and oxidative stress. Studies demonstrate that heat exposure leads to significant rises in H_2_O_2_ and O_2_^-^ levels, though notable interspecific variation. For instance, H_2_O_2_ increases by approximately 245% in some species, while barley, maize, and tomato cultivars exhibit a more moderate increase of around 50% (Zhao et al., 2018; Ahammed et al., 2019; Ayub et al., 2021; Zahra et al., 2022). Similarly, O_2_^-^ levels surge by 200% in rice, tomato, and maize cultivars (**Table 7**) (Zhao et al., 2018; Zhang et al., 2019; Jahan et al., 2022). However, contrasting reports indicate decreases in O_2_^-^ and H_2_O_2_ by 26% and 27%, respectively, in barley shoots under heat stress (Zhanassova et al., 2021). These discrepancies may stem from differences in experimental conditions, as mentioned previously. Such variations emphasize the complexity of oxidative stress responses and underscore the importance of standardized experimental approaches when comparing interspecific ROS regulation under heat stress, and highlight the need for further investigation into oxidative stress responses.

**Table 7:** Changes in reactive oxygen species and malondialdehyde levels in different species of plants’ response to heat stress.

Plant species	Changes in %	T°C	Duration of heat stress treatment	References
Superoxide (O_2_^-^)				
*Hordeum vulgare*	~27% ↓	40°C	5 days	Zhanassova et al., 2021
*Oryza sativa*	~199% ↑	38°C	3 days	Zhao et al., 2018
*Zea mays*	~200% ↑	40°C	5 days	Zhang et al., 2019
*Solanum lycopersicum*	~200% ↑	38°C	7 days	Jahan et al., 2022
Hydrogen peroxide (H_2_O_2_)				
*Hordeum vulgare*	~50% ↑	42°C	2 days	Zahra et al., 2022
	~26% ↓	40°C	5 days	Zhanassova et al., 2021
*Oryza sativa*	~245% ↑	38°C	3 days	Zhao et al., 2018
*Zea mays*	~50% ↑	40°C	6 hours	Ayub et al., 2021
*Solanum lycopersicum*	65% ↑	40°C	9 hours	Ahammed et al., 2019
Malondialdehyde (MDA)				
*Hordeum vulgare*	~100% ↑	42°C	2 days	Zahra et al., 2022
*Oryza sativa*	~151% ↑	38°C	3 days	Zhao et al., 2018
*Zea mays*	~100% ↑	40°C	5 days	Zhang et al., 2019
*Solanum lycopersicum*	~150-200% ↑	40°C	9 hours	Ahammed et al., 2019
*Arabidopsis thaliana*	~65% ↑	28°C	3 days	Jin et al., 2011

Arrows’ heads up (↑) represent positive changes, and down (↓) represent negative changes. ~ indicates approximately. T°C, temperature in Celsius.

A major consequence of excessive ROS accumulation is lipid peroxidation, which compromises membrane integrity. Malondialdehyde (MDA), a lipid peroxidation byproduct, serves as a reliable biomarker for oxidative membrane damage. Under heat stress, MDA levels increase by 151% and 150% in rice and tomato cultivars, respectively, with the most pronounced changes observed in rice cultivars (XQZ and G46) during the reproductive stage (Zhao et al., 2018; Ahammed et al., 2019). Comparatively, barley, *Arabidopsis*, and maize cultivars show increases of 100%, 65%, and 100%, respectively (**Table 7**), confirming MDA as a consistent indicator of cellular damage across species (Jin et al., 2011; Zhang et al., 2019; Zahra et al., 2022).

The threat posed by ROS accumulation triggers diverse biochemical defense mechanisms to counteract oxidative stress (Gill and Tuteja, 2010; Fortunato et al., 2023; Li et al., 2023; Nurbekova et al., 2024; Jardim-Messeder et al., 2025; Kračun et al., 2025). While some species enhance their antioxidant capacity, others exhibit reduced efficacy, underscoring the need for detailed analysis of enzymatic and non-enzymatic systems in maintaining cellular homeostasis under heat stress (Foyer and Noctor, 2013; Noctor et al., 2018; Fortunato et al., 2023).

### 4.2 Antioxidant defense systems

To mitigate ROS-induced damage, plants activate both enzymatic and non-enzymatic antioxidant systems (Foyer and Noctor, 2013; Noctor et al., 2018; Bhuyan et al., 2019; Jardim-Messeder et al., 2025; Jiang et al., 2025; Yang et al., 2025). Key enzymatic antioxidants include superoxide dismutase (SOD), catalase (CAT), peroxidases (POD), and ascorbate peroxidase (APX), which collectively scavenge excess ROS and protect cellular structures (Ru et al., 2023; Jiang et al., 2025; Rao and Zheng, 2025; Zhou et al., 2025). Non-enzymatic antioxidants such as ascorbate and glutathione (GSH) further contribute to redox homeostasis and stress tolerance (Noctor et al., 2018; Jardim-Messeder et al., 2025; Msarie et al., 2025; Rao and Zheng, 2025). The activation of these defense mechanisms varies significantly among plant species (**Table 8**).

**Table 8:** Changes in enzymatic and non-enzymatic antioxidant components in different species of plants’ response to heat stress.

Plant species	Changes in %	T°C	Duration of heat stress treatment	References
Superoxide dismutase (SOD)				
*Hordeum vulgare*	~205% ↑	35°C	7 days	Hussain et al., 2019b
*Oryza sativa*	~50% ↓	38°C	5 days	Liu et al., 2019
*Zea mays*	40-45% ↓	40°C	20 mins	Doğru, 2021
	50% ↑	41°C	6 hours	Hussain et al., 2016
*Solanum lycopersicum*	305% ↑	40°C	9 hours	Ahammed et al., 2019
*Arabidopsis thaliana*	~500% ↓	38°C	6 hours	Wang et al., 2020
Catalase (CAT)				
*Hordeum vulgare*	~127% ↑	35°C	7 days	Hussain et al., 2019b
*Oryza sativa*	~50% ↓	38°C	5 days	Liu et al., 2019
*Zea mays*	~20% ↓	38°C	15 days	Hussain et al., 2019a
*Solanum lycopersicum*	~50% ↑	38°C	3 days	Ding et al., 2018
*Arabidopsis thaliana*	~400% ↑	38°C	6 hours	Wang et al., 2020
Peroxidase (POD)				
*Hordeum vulgare*	~128% ↑	35°C	7 days	Hussain et al., 2019b
*Oryza sativa*	~32.1% ↓	38°C	5 days	Liu et al., 2019
*Zea mays*	~10% ↓	38°C	15 days	Hussain et al., 2019a
*Solanum lycopersicum*	~100% ↑	38°C	3 days	Ding et al., 2018
*Arabidopsis thaliana*	~250% ↑	38°C	6 hours	Wang et al., 2020
Ascorbate peroxidase (APX)				
*Oryza sativa*	~32.1% ↓	38°C	5 days	Liu et al., 2019
*Zea mays*	~50% ↓	38°C	15 days	Hussain et al., 2019a
*Solanum lycopersicum*	28% ↑	40°C	9 hours	Ahammed et al., 2019
Glutathione (GSH)				
*Zea mays*	28% ↑	38°C	15 days	Hussain et al., 2019a
*Solanum lycopersicum*	~150% ↑	38°C	3 days	Ahammed et al., 2019

Arrows’ heads up (↑) represent positive changes, and down (↓) represent negative changes. ~ indicates approximately. T°C, temperature in Celsius.

**Figure 3:**
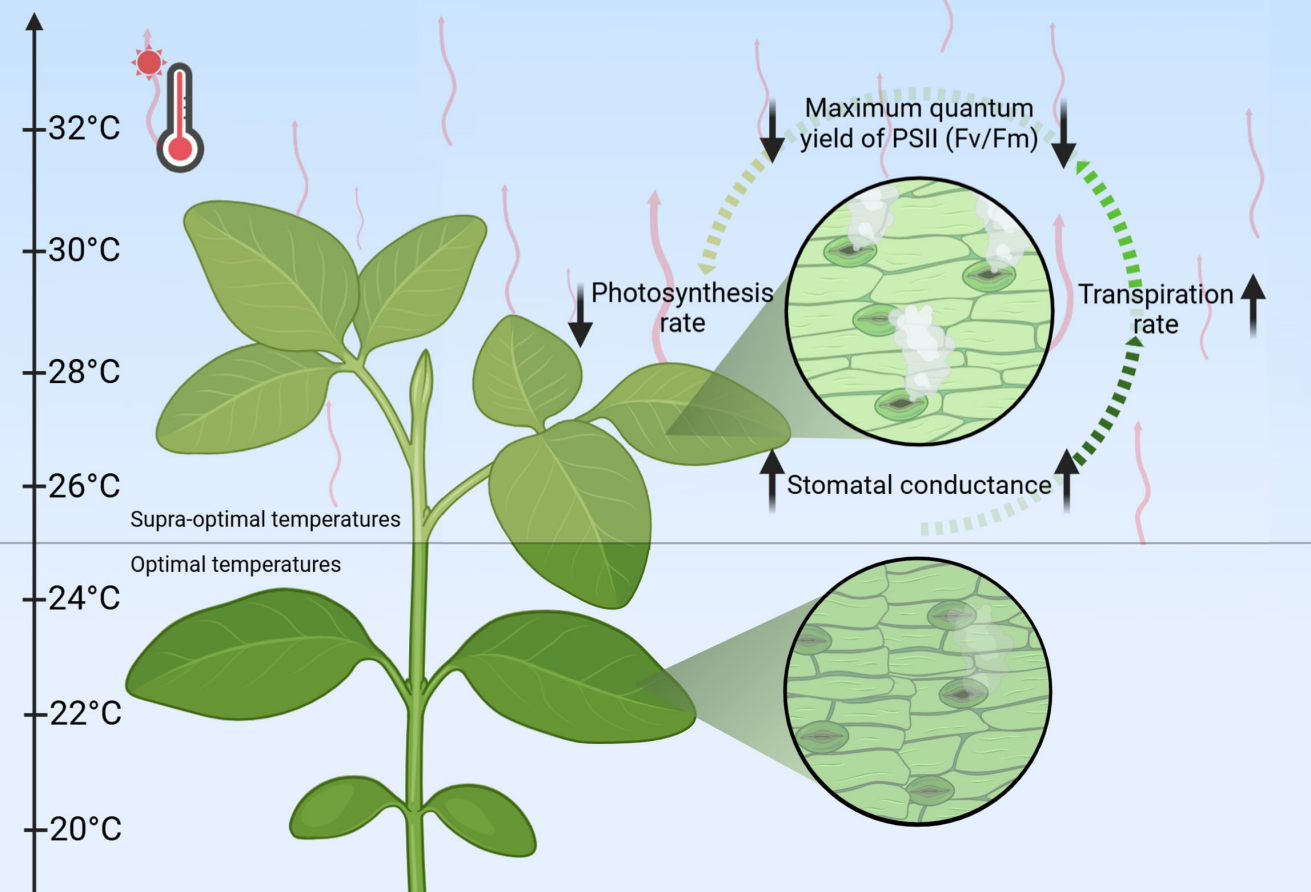
Effect of heat stress on ROS accumulation and antioxidant defense and restoration system of plants. Arrows with dotted ends show the transition between plant states (red - deterioration, green - improvement); Arrow with a blunted-end head represents the inhibitory effect of the antioxidant system on oxidative damage; SOD, Superoxide dismutase; POD, Peroxidase; APX, Ascorbate peroxidase; GSH, Glutathione.

While ROS accumulation under heat stress is well-documented, the efficiency of antioxidant responses shows remarkable interspecific variation (Gill and Tuteja, 2010; Foyer and Noctor, 2013; Noctor et al., 2018; Fortunato et al., 2023; Li et al., 2023; Nurbekova et al., 2024). Some plants exhibit strong activation of both enzymatic and non-enzymatic antioxidants, effectively mitigating oxidative damage (**Figure 3**), while others show reduced antioxidant capacity, increasing their vulnerability to stress-induced cellular injury (Foyer and Noctor, 2013; Noctor et al., 2018; Bhuyan et al., 2019). These findings reveal a spectrum of defense strategies among species, with some having evolved more effective mechanisms to maintain redox homeostasis. The equilibrium between ROS generation and antioxidant defense is therefore pivotal in determining a plant’s ability to withstand thermal stress and maintain physiological function (Foyer and Noctor, 2013; Noctor et al., 2018). A comprehensive understanding of these biochemical adaptations is essential for developing targeted strategies to enhance crop resilience through breeding and biotechnological approaches.

## 5 Mitigation strategies against heat stress

Heat stress significantly impairs agricultural crops by disrupting physiological processes and hindering growth and development. Germination rates can decline by up to 95%, while shoot and root growth reductions range from 3.2% to 70% (**Tables 1**, **2**). These morphological changes are accompanied by adaptive physiological and biochemical responses, which may themselves have detrimental effects. To address these challenges, biotechnological and agronomic strategies for enhancing thermotolerance are critical. Promising approaches include the pre-sowing seed priming technique, post-germination supplementary treatments, microbial biostimulation, and microRNA manipulation.

### 5.1 Pre-sowing seed priming for enhanced plant thermotolerance

Pre-sowing seed priming has emerged as a promising agricultural technique to enhance plant resilience to temperature fluctuations (Filippou et al., 2013; Pagano et al., 2023). This method involves controlled seed treatment to initiate crucial metabolic processes prior to radicle emergence, effectively ‘preparing seeds for faster, more synchronized germination under favorable conditions. Primed plants exhibit superior survival rates under extreme conditions compared to non-primed counterparts (Hönig et al., 2023; Pagano et al., 2023). This method enhances both seed germination and crop productivity through various approaches, including osmopriming, hydropriming, halopriming, and nutrient priming. Moderate temperature exposure has also been shown to boost heat tolerance, primarily by stabilizing the photosynthetic apparatus (Wahid et al., 2008; Ru et al., 2023).

Drought priming, in particular, improves thermotolerance by enhancing morphological parameters and photosynthetic efficiency (**Table 9**). These benefits are mediated through molecular adaptations, as demonstrated in maize cultivars where thermopriming significantly increases activity of ROS-scavenging enzymes (SOD, APX, POD, CAT), thereby reducing oxidative damage as indicated by lower MDA levels (Ru et al., 2023). Similarly, cold stress priming (0 °C) improves germination rates in barley cultivars (Mei and Song, 2010). The efficacy of priming is closely tied to stress memory mechanisms, where initial mild stress induces epigenetic modifications that enable more robust responses to subsequent stress events (Li and Gong, 2011; Walter et al., 2013; Antoniou et al., 2016). This priming-induced memory represents a powerful tool for developing climate-resilient crops in the face of increasing environmental variability.

**Table 9:** Pre-sowing seed physical and chemical priming enhances thermotolerance in various plants.

Priming source	Plant species	Changes	T°C	Duration of heat stress treatment	References
Physical					
Cold 0 °C (1–4 days)	*Hordeum vulgare* cv. Pijiu	Germination rate increased by 55% ↑	40°C	2 days	Mei and Song, 2010
Artificial drought priming (6 days)	*Zea mays* cv. Zhengdan 958	Relative growth rate increased by 29% ↑	36°C	6 days	Ru et al., 2023
Chemical					
Hydrogen peroxide (150µM)	*Zea mays* cv. SWL-2002	Germination rate increased by 131.5% ↑	42°C	24 hours	Wahid et al., 2008
Spermidine (1.5mM)	*Oryza sativa L. ssp. indica* cv. YLY 689	Germination rate increased by 147.4% ↑	40°C	3 days	Fu et al., 2019
		Shoot dry weight increased by 121.1% ↑			
Salicylic acid (140 mg L−1)	*Oryza sativa* cv. Dhan 84	Shoot dry weight increased by 8% ↑	37.1 ± 1.7°C	30-60–90 days	Ahmed et al., 2024
Chitosan (100 mg L−1)		Shoot dry weight increased by 7.5% ↑			

Arrows’ heads up (↑) represent positive changes. T°C, temperature in Celsius.

H_2_O_2_ is a cost-effective and widely used priming agent that promotes root/shoot growth and seed germination under heat stress. Its ability to enhance these morphological parameters and germination relies on its capacity to modulate key cellular processes. In maize, H_2_O_2_ pretreatment enhances photosynthetic efficiency while reducing ROS and MDA concentrations (Wahid et al., 2008). Additionally, it also activates heat stress proteins (27 and 63 kDa) that regulate membrane integrity and structure during root and shoot development, providing molecular support crucial for overall growth (Schoffl et al., 1999; Wahid et al., 2008).

Combined salicylic acid (SA) and chitosan pre-sowing seed treatment enhances rice hybrids’ growth parameters under heat stress, enhancing morphology, photosynthetic performance, and antioxidant defense while reducing ROS and MDA levels (Ahmed et al., 2024). SA alone boosts photosynthetic rate, stomatal conductance, and transpiration rate in rice, though the combined treatment offers superior protection (Ahmed et al., 2024). Notably, unprimed plants are more sensitive to heat during the vegetative stage than during the flowering stages, as seen in gerbera (transpiration rates) and hot pepper (photosynthetic efficiency related to membrane thermostability) (Rajametov et al., 2021; Yang et al., 2023).

SA priming also prevents heat-induced inhibition of RuBisCo, a critical enzyme for carbon fixation, thereby maintaining photosynthetic efficiency comparable to heat-tolerant cultivars (Wang et al., 2010a; Ahmed et al., 2024). By maintaining RuBisCo activity, primed plants exhibit photosynthetic efficiency comparable to heat-tolerant cultivars (Qin et al., 2025; Zheng et al., 2025). This molecular mechanism directly underpins the improved growth and productivity observed in chemically primed plants under heat stress. Likewise, spermidine-treated rice cultivars demonstrate reduced ROS and MDA levels, leading to improved shoot growth and overall plant development under high-temperature conditions (Fu et al., 2019).

Priming activates ROS-scavenging enzymes, heat shock proteins (HSPs), and molecular chaperones, enhancing abiotic stress tolerance in crops like pepper, maize, soybean, spinach, and wheat (Iqbal and Ashraf, 2007; Farooq et al., 2008; Korkmaz and Korkmaz, 2009; Zhuo et al., 2009; Chen et al., 2010). Chemical priming agents, including H_2_O_2_, abscisic acid (ABA), and SA, improve resilience by modulating photosynthesis, ROS detoxification pathways, and MG metabolism (de Azevedo Neto et al., 2005; Chao et al., 2009; Liu et al., 2010; Wang et al., 2010a, b; Hasanuzzaman et al., 2011; Ishibashi et al., 2011; Gondim et al., 2012; Mostofa and Fujita, 2013; Mostofa et al., 2014; Sathiyaraj et al., 2014; Teng et al., 2014; Ahmed et al., 2024). Hormonal regulation plays a key role, with heat stress increasing ABA while decreasing gibberellic acid (GA) and indole-3-acetic acid (IAA) levels, leading to reproductive impairment (Shimizu and Kuno, 1967; Nakajima et al., 1991; Tang et al., 1996; El-Maarouf-Bouteau et al., 2013; Ishibashi et al., 2015, 2017; Kadyrbaev et al., 2021; Hirayama and Mochida, 2022). These intricate molecular and hormonal interactively collectively contribute to the observable improvements in plant morphology and overall thermotolerance. The synergistic effects of these mechanisms enhance plants’ capacity to maintain physiological function under elevated temperatures. Understanding these hormonal interactions may further refine priming strategies to further enhance plant thermotolerance and improve crop productivity under extreme environmental conditions.

### 5.2 Post-Germination chemical and nutritional treatments for heat stress mitigation

Beyond pre-sowing seed priming, post-germination treatments provide a valuable complementary approach to enhance thermotolerance in developing plants. The exogenous applications of plant growth regulators, essential nutrients, and protective compounds has demonstrated significant efficacy in maintaining key morphological parameters – including shoot/root growth, floral development, and fruit weight (Zhao et al., 2018; Murad Lima et al., 2025; Ssemugenze et al., 2025; Ali et al., 2021; Khan et al., 2024). While elevated temperatures typically impair photosynthetic efficiency (**Tables 5**, **6**), these supplemental treatments effectively mitigate such physiological limitation during vegetative growth (**Table 10**). Notably, foliar application of exogenous SA increases chlorophyll content and relative water content (RWC) by approximately 50% in barley while similarly improving growth rates in maize (Khanna et al., 2016; Zahra et al., 2022). The protective mechanisms involve both direct physiological effects and the upregulation of antioxidant defense systems, as evidenced by enhanced activity of ROS-scavenging enzymes (Khanna et al., 2016; Hussain et al., 2019b; Ali et al., 2021; Raghunath et al., 2021; Jahan et al., 2022; Zahra et al., 2022). These coordinated responses collectively strengthen plant resilience to thermal stress while maintaining productivity.

**Table 10:** Post-Germination chemical and nutritional treatments enhance thermotolerance in various plants.

Treatment source and conditions	Plant species	Changes	T°C	Duration of heat stress treatment	References
Sulfur 2, 4, 6, and 8 ppm (6 ppm effective concentration)	*Solanum lycopersicum L.* genotypes, “Roma” (thermotolerant) and “Ahmar” (thermosensitive)	Increased growth rate ~77% ↑	45 ± 2°C	20 days	Ali et al., 2021
Sodium silicate 1.5 mM Si (Sprayed)	*Hordeum vulgare L. cvr. Jow-83 and B-12026*	Increased growth rate by ~40% ↑	35/30 ± 2°C	7 days	Hussain et al., 2019b
Salicylic acid 2 and 5mM	*Hordeum vulgare L. cvr Jau-87, B-10007, B-14003, B-14037*	Chlorophyll, carotenoid, and RWC increased by ~50% ↑	42°C	24 hours	Zahra et al., 2022
Salicylic acid 10-800 μM (<400 μM affects positively)	*Zea mays L. cvr. not shown*	Increased growth rate >50% ↑	40°C	2 hours	Khanna et al., 2016
Putrescine 1 mM	*Solanum lycopersicum L. (cvr. not shown)*	Increased antioxidant enzymes: POD -32% ↑, CAT -38% ↑, APX -35% ↑	38°C	7 days	Jahan et al., 2022
Melatonin 25 µM (field experiments)	*Solanum lycopersicum L.* Heat tolerant (T60 F1 and Super cash F1) Heat sensitive (*Naqeeb* and *Nagina*)	Increased morphological parameters, including a growth rate of ~30-50% ↑	41/5°C	40 days	Khan et al., 2024
Gibberellic acid (GA3) 50 ppm (field experiment)	*Oryza sativa L cvr* *Uma (MO-16)*	Reproductive organ growth increased by ~50% ↑	40°C	113 days	Raghunath et al., 2021
		Photosynthesis-related increased ~20% ↑			
Brassinosteroid (BR) 5 ppm (field experiment)		Increased plant height and panicle length ↑			

Arrows’ heads up (↑) represent positive changes. **~** indicates approximately. > indicates more than. T°C, temperature in Celsius.

Brassinosteroids post-germination treatments also contribute to heat stress mitigation by promoting reproductive organ growth and enhancing photosynthesis-related markers. In contrast, GA has a stronger impact on morphological traits, such as increasing plant height in rice exposed to 40 °C (Raghunath et al., 2021). Putrescine, a spermidine/spermine precursor, protects plants by regulating potassium channels in guard cells, improving membrane stability. In tomatoes, exogenous putrescine application in the vegetative growth stage activates ROS-scavenging enzymes, increases photosynthetic pigments, suppresses oxidative stress markers, and upregulates HSP 70 and HSP 90 (Jahan et al., 2022).

Melatonin, structurally related to putrescine, enhances shoot/leaf growth while improving tomato thermotolerance when applied as a foliar spray (Khan et al., 2024). Recent studies have highlighted the role of silicon and silicon-containing compounds in stress mitigation as post-germination treatment agents. These molecules stabilize lipid membranes, enhance thermal stability, and promote polysaccharide accumulation, boosting heat resilience (Swain and Rout, 2017; Kumar et al., 2023). Sodium silicate treatment increases barley growth rates by 40% compared to untreated plants (Hussain et al., 2019b).

In addition to these compounds, essential minerals like nitrogen, sodium, and sulfur play a vital role in stress mitigation. Sulfur, crucial for protein biosynthesis and antioxidant defenses, can increase tomato growth rate by 77% under heat stress as it is a central component of thiol groups involved in plant stress responses (Bashir et al., 2015; Ihsan et al., 2019; Ali et al., 2021). While abiotic stress priming and chemical treatments are widely used, biotic stress priming, such as beneficial microbial and viral colonies, also positively influences plant thermotolerance (Khan et al., 2022).

### 5.3 Microbial biostimulation for heat stress mitigation

The plant microbiome provides additional defense against biotic stressors by enhancing tolerance through a mutualistic relationship (Chauhan et al., 2023; Teiba et al., 2024). Though direct evidence of microbial biostimulation in major crops remains limited, certain bacterial strains support plant growth under heat stress (**Table 11**). For instance, root-associated bacteria protect tomatoes, while endophytic fungi promote resistance and normal growth (Cameron et al., 2013; Pieterse et al., 2014; Narendra Babu et al., 2015).

**Table 11:** Microbial biostimulation to enhance thermotolerance in plant species.

Treating organism	Plants species	Changes	T°C	Duration of heat stress treatment	References
Fungi					
*Paecilomyces formosus*	*Oryza sativa L. (Dongjin)*	Increased growth rate ↑	30°C	10 days	Waqas et al., 2015
*Septoglomus constrictum*	*Solanum lycopérsicum (var. MoneyMaker)*	Increased growth rate ↑	42°C	6h (10 days)	Duc et al., 2018
*Serendipita indica*	*A. thaliana Col-0*	Increased shoot length and weight ↑	28°C	7–14 days	Chen et al., 2022
Bacteria					
*Bacillus cereus*	*Solanum lycopérsicum (Vir Yegwang)*	Increased growth rate ↑	37°C	15 days	Khan et al., 2020
*Bacillus safensis*	*Solanum lycopersicum L (Riogrande)*	Increased growth rate ↑	42°C	5 hours per day	Mukhtar et al., 2023
*Bacillus cereus*	*Solanum lycopérsicum (cv. Riogrande)*	Increased growth rate ↑	42°C	6 h/day till the fruiting stage.	Mukhtar et al., 2020

Arrows’ heads up (↑) represent positive changes. **~** indicates approximately. T°C, temperature in Celsius.

One notable example is *Paecilomyces formosus*, a fungal species that enhances rice growth under normal and high temperatures, likely through secondary metabolite production (Waqas et al., 2015). It also modulates ABA and jasmonic acid (JA) levels, similar to its effects in *Capsicum annuum L.* and *Dichanthelium lanuginosum* (Redman et al., 2011; Khan et al., 2015; Waqas et al., 2015). Fungi like *Rhizophagus irregularis* and *Funneliformis mosseae* improve maize photosynthetic efficiency (chlorophyll content, fluorescence, rate) while reducing MDA levels through improved N/Mg uptake (Mathur et al., 2021). In tomatoes, *Septoglomus constrictum* and *Septoglomus deserticola* enhance morphology and reduce oxidative stress at 42 °C, though without photosynthetic improvements (Duc et al., 2018). *Serendipita indica* similarly benefits *Arabidopsis* morphology under heat stress (Chen et al., 2022).

The plant microbiome provides additional defense against biotic stressors by enhancing tolerance through a mutualistic relationship (Chauhan et al., 2023; Teiba et al., 2024). Though direct evidence of microbial biostimulation in major crops remains limited, certain bacterial strains support plant growth under heat stress (**Table 11**). For instance, root-associated bacteria protect tomatoes, while endophytic fungi promote resistance and normal growth (Cameron et al., 2013; Pieterse et al., 2014; Narendra Babu et al., 2015).

### 5.4 Mitigation against heat by regulating miRNA

miRNAs (20–24 nt) are emerging tools for improving stress resilience in crops by post-transcriptionally regulating target genes (Chen, 2009; Voinnet, 2009; Asefpour Vakilian, 2020; Ding et al., 2020; Chaudhary et al., 2021; Raza et al., 2021). Furthermore, miRNAs have potential applications in genetic engineering, where their manipulation can improve crop quality and create hybrids with enhanced heat tolerance (Zheng et al., 2025; Zolkiewicz and Gruszka, 2025). Our analysis has identified several miRNAs associated with morphological and physiological changes under heat stress. Moreover, targeted miRNA modifications can contribute to the development of thermotolerant plant hybrids (**Table 12**).

**Table 12:** MicroRNAs and their target genes in heat stress response.

miRNA	Target Genes	Plants species Temperature in °C Duration of heat stress treatment	Role/Effect on Heat Stress	References
miR-398	CSD1, CSD2 (copper superoxide dismutase)	*Arabidopsis thaliana* (*SALK_024857*) (*SALK_025986*) 37°C 2 hours	Suppresses CSD1 and CSD2 expression, leading to Increased heat tolerance through ROS management and upregulation of HSPs	Guan et al., 2013
miR-172	EAT1 (TOE1), TOE2, and SCHLAFMUTZE (SMZ) FT (flowering time) increased	*Arabidopsis thaliana* (Col-0) 28°C 4–6 days	Delaying the transition to a flowering stage helps plants better cope with heat stress because at vegetative stress, plants are more tolerant than at the flowering stage.	May et al., 2013
miR-160	ARF17 and ARF13	*Hordeum vulgare L. (cv Rolap)* 35.5°C 3, 6, 24 hours	miR-160 resistance is capable of increasing auxin generation, which affects the thermotolerance of plants	Kruszka et al., 2014
miR-160a			Delays cell proliferation and flower production by regulating the auxin pathway, affecting growth under stress. Furthermore, the knockdown of miR-160a led to an increase in auxin production	
miR156	SPL transcription factor	*Arabidopsis thaliana* (Col-0) 37°C 1 hour	Overexpression of miR156 is capable of increasing heat stress memory	Stief et al., 2014

One key miRNA involved in heat stress response is miR-398, which is upregulated under high-temperature conditions. Under heat stress, miR-398 upregulation suppresses the expression of the copper superoxide dismutase (CSD1/CSD2) while increasing heat shock protein (HSP) expression, improving *Arabidopsis* thermotolerance (Sunkar and Zhu, 2004; Sunkar et al., 2006; Chen, 2009; Kaczkowski et al., 2009; Guan et al., 2013; Zhao et al., 2016). miR-160 overexpression similarly enhances heat tolerance in *Arabidopsis* by regulating HSPs and plant development (Lin et al., 2018).

miRNAs also regulate flowering time under heat stress (Li et al., 2022). The transition from the vegetative phase to flowering is controlled by SQUAMOSA PROMOTER BINDING PROTEIN-LIKE (SPL) and APETALA2 (AP2) genes (Stief et al., 2014; Gahlaut et al., 2018; Ma et al., 2020). miR-156 and miR-172 regulate SPL expression, with elevated miR-172 levels (mediated by miR-156) under high-temperature stress leading to reduced SPL expression. This downregulation of SPL subsequently activates FLOWERING LOCUS T (FT), establishing a link between temperature sensing and flowering time (Wu et al., 2009; Kim et al., 2012; Cui et al., 2014; Stief et al., 2014). Additionally, miR-166 overexpression inhibits PHAVOLUTA and REVOLUTA, two genes responsible for leaf development, as well as HOX9, a member of the HD-Zip family (Barik et al., 2014). Similarly, miR-160a downregulates ARF17 and ARF13, affecting the auxin signaling pathway, delaying development, cell proliferation, and flowering (Barik et al., 2014; Kruszka et al., 2014). This delay, together with miR-166’s disruption of leaf development, enhances *Arabidopsis thaliana’s* tolerance to high temperatures (Reinhart et al., 2002; Mallory et al., 2005; Barik et al., 2014). By precisely regulating stress-response pathways, miRNAs offer powerful biotechnological tools for developing heat-tolerant crops through targeted gene manipulation (Batool et al., 2025; Gao et al., 2025; Spychała et al., 2025; Zheng et al., 2025).

## 6 Conclusion

Climate change and global population growth pose a severe threat to agricultural sustainability, with rising temperatures particularly diminishing crop yields. High-temperature stress has a negative impact on plant growth and development. While extensive research exists, much of it focuses on individual species or isolated mechanisms, lacking broader comparative insights. This review systematically compared heat stress responses and mitigation strategies across four economically important crops - barley, maize, rice, and tomato - alongside *Arabidopsis thaliana*. Morphological analysis revealed that root parameters are more sensitive indicators of thermal susceptibility than shoot parameters, though germination rate changes primarily occur under extreme heat. Physiologically, all studied species exhibited consistent reductions in photosynthetic rates and PSII efficiency (Fv/Fm), while stomatal conductance and transpiration rates varied by species and stress duration. Biochemical analyses of ROS accumulation and antioxidant activity presented complex, mixed results - upregulation and downregulation, underscoring the need for deeper investigation into oxidative stress pathways. Among mitigation strategies, priming emerged as an effective one due to its low cost and practical applicability. Other promising approaches include chemical/nutritional treatments, microbial biostimulants, and miRNA-based regulation. Notably, miRNA manipulation offers significant potential for developing thermotolerant crop varieties, promising advancement in both scientific research and agricultural applications.

## References

[B1] Abo Gamar M. I. Kisiala A. Emery R. J. N. Yeung E. C. Stone S. L. Qaderi M. M. (2019). Elevated carbon dioxide decreases the adverse effects of higher temperature and drought stress by mitigating oxidative stress and improving water status in Arabidopsis thaliana. Planta 250, 1191–1214. doi: 10.1007/s00425-019-03213-3, PMID: 31190116

[B2] Afshar T. Dass N. Lau C. Lee A. (2016). The effect of temperature on the germination of Arabidopsis thaliana seeds. Expedition 6, 1–11.

[B3] Ahammed G. J. Xu W. Liu A. Chen S. (2019). Endogenous melatonin deficiency aggravates high temperature-induced oxidative stress in Solanum lycopersicum L. Environ. Exp. Bot. 161, 303–311. doi: 10.1016/j.envexpbot.2018.06.006

[B4] Ahmed S. Ahmed S. F. Biswas A. Sultana A. Issak M. (2024). Salicylic acid and chitosan mitigate high temperature stress of rice via growth improvement, physio-biochemical adjustments and enhanced antioxidant activity. Plant Stress 11, 100343. doi: 10.1016/j.stress.2023.100343

[B5] Ali M. M. Waleed Shafique M. Gull S. Afzal Naveed W. Javed T. Yousef A. F.. (2021). Alleviation of heat stress in tomato by exogenous application of sulfur. Horticulturae 7, 24. doi: 10.3390/horticulturae7020021

[B6] Antoniou C. Savvides A. Christou A. Fotopoulos V. (2016). Unravelling chemical priming machinery in plants: the role of reactive oxygen–nitrogen–sulfur species in abiotic stress tolerance enhancement. Curr. Opin. Plant Biol. 33, 101–107. doi: 10.1016/j.pbi.2016.06.020, PMID: 27419886

[B7] Asefpour Vakilian K. (2020). Machine learning improves our knowledge about miRNA functions towards plant abiotic stresses. Sci. Rep. 10, 3041. doi: 10.1038/s41598-020-59981-6, PMID: 32080299 PMC7033123

[B8] Ayub M. Ashraf M. Y. Kausar A. Saleem S. Anwar S. Altay V.. (2021). Growth and physio-biochemical responses of maize (Zea mays L.) to drought and heat stresses. Plant Biosyst. 155, 535–542. doi: 10.1080/11263504.2020.1762785

[B9] Baloch R. Saleem M. F. Shahbaz M. Sarwar M. (2024). Evaluation of basmati rice (Oryza sativa L.) genotypes for seedling growth and leaf physiology under high-temperature stress. J. Agron. Crop Sci. 210, e12777. doi: 10.1111/jac.12777

[B10] Barik S. SarkarDas S. Singh A. Gautam V. Kumar P. Majee M.. (2014). Phylogenetic analysis reveals conservation and diversification of micro RNA166 genes among diverse plant species. Genomics 103, 114–121. doi: 10.1016/j.ygeno.2013.11.004, PMID: 24275521

[B11] Barten R. Kleisman M. D’Ermo G. Nijveen H. Wijffels R. H. Barbosa M. J. (2022). Short-term physiologic response of the green microalga Picochlorum sp. (BPE23) to supra-optimal temperature. Sci. Rep. 12, 3290. doi: 10.1038/s41598-022-06954-6, PMID: 35228560 PMC8885816

[B12] Bashir H. Ibrahim M. M. Bagheri R. Ahmad J. Arif I. A. Baig M. A.. (2015). Influence of sulfur and cadmium on antioxidants, phytochelatins and growth in Indian mustard. AoB Plants 7, plv001. doi: 10.1093/aobpla/plv001, PMID: 25587194 PMC4323519

[B13] Baskin C. C. Baskin J. M. (2000). Seeds: ecology, biogeography, and, evolution of dormancy and germination. (New York: Academic Press).

[B14] Batool I. Ayyaz A. Qin T. Wu X. Chen W. Hannan F.. (2025). Morphological, physiological, and molecular responses to heat stress in Brassicaceae. Plants 14, 152. doi: 10.3390/plants14020152, PMID: 39861509 PMC11768255

[B15] Benitez-Alfonso Y. Soanes B. K. Zimba S. Sinanaj B. German L. Sharma V.. (2023). Enhancing climate change resilience in agricultural crops. Curr. Biol. 33, R1246–R1261. doi: 10.1016/j.cub.2023.10.028, PMID: 38052178

[B16] Bernacchi C. J. Ruiz-Vera U. M. Siebers M. H. DeLucia N. J. Ort D. R. (2023). Short- and long-term warming events on photosynthetic physiology, growth, and yields of field grown crops. Biochem. J. 480, 999–1014. doi: 10.1042/BCJ20220433, PMID: 37418286 PMC10422931

[B17] Bhuyan M. H. M. B. Hasanuzzaman M. Mahmud J. A. Hossain M. Bhuiyan T. F. Fujita M. (2019). Unraveling Morphophysiological and Biochemical Responses of Triticum aestivum L. @ to Extreme pH: Coordinated Actions of Antioxidant Defense and Glyoxalase Systems. Plants 8, 24. doi: 10.3390/plants8010024, PMID: 30669317 PMC6359243

[B18] Brändel M. (2004). The role of temperature in the regulation of dormancy and germination of two related summer-annual mudflat species. Aquat. Bot. 79, 15–32. doi: 10.1016/j.aquabot.2003.11.008

[B19] Brunel-Muguet S. d’Hooghe P. Bataillé M.-P. Larré C. Kim T.-H. Trouverie J.. (2015). Heat stress during seed filling interferes with sulfur restriction on grain composition and seed germination in oilseed rape (Brassica napus L.). Front. Plant Sci. 6. doi: 10.3389/fpls.2015.00213, PMID: 25914702 PMC4392296

[B20] Burroughs C. H. Montes C. M. Moller C. A. Mitchell N. G. Michael A. M. Peng B.. (2023). Reductions in leaf area index, pod production, seed size, and harvest index drive yield loss to high temperatures in soybean. J. Exp. Bot. 74, 1629–1641. doi: 10.1093/jxb/erac503, PMID: 36571807

[B21] Cameron D. D. Neal A. L. van Wees S. C. M. Ton J. (2013). Mycorrhiza-induced resistance: more than the sum of its parts? Trends Plant Sci. 18, 539–545. doi: 10.1016/j.tplants.2013.06.004, PMID: 23871659 PMC4194313

[B22] Challinor A. J. Watson J. Lobell D. B. Howden S. M. Smith D. R. Chhetri N. (2014). A meta-analysis of crop yield under climate change and adaptation. Nat. Climate Change 4, 287–291. doi: 10.1038/nclimate2153

[B23] Chao Y.-Y. Hsu Y. T. Kao C. H. (2009). Involvement of glutathione in heat shock– and hydrogen peroxide–induced cadmium tolerance of rice (Oryza sativa L.) seedlings. Plant Soil 318, 37–45. doi: 10.1007/s11104-008-9815-x

[B24] Chaudhary S. Grover A. Sharma P. C. (2021). MicroRNAs: potential targets for developing stress-tolerant crops. Life 11, 289. doi: 10.3390/life11040289, PMID: 33800690 PMC8066829

[B25] Chauhan P. Sharma N. Tapwal A. Kumar A. Verma G. S. Meena M.. (2023). Soil microbiome: diversity, benefits and interactions with plants. Sustainability 15, 14643. doi: 10.3390/su151914643

[B26] Chen X. (2009). Small RNAs and their roles in plant development. Annu. Rev. Cell Dev. Biol. 25, 21–44. doi: 10.1146/annurev.cellbio.042308.113417, PMID: 19575669 PMC5135726

[B27] Chen K. Arora R. Arora U. (2010). Osmopriming of spinach (Spinacia oleracea L. cv. Bloomsdale) seeds and germination performance under temperature and water stress. Seed Sci. Technol. 38, 36–48. doi: 10.15258/sst.2010.38.1.04

[B28] Chen X.-J. Yin Y.-Q. Zhu X.-M. Xia X. Han J.-J. (2022). High ambient temperature regulated the plant systemic response to the beneficial endophytic fungus Serendipita indica. Front. Plant Sci. 13. doi: 10.3389/fpls.2022.844572, PMID: 35371134 PMC8966885

[B29] Cui L.-G. Shan J.-X. Shi M. Gao J.-P. Lin H.-X. (2014). The miR156-SPL-9-DFR- pathway coordinates the relationship between development and abiotic stress tolerance in plants. Plant J. 80, 1108–1117. doi: 10.1111/tpj.12712, PMID: 25345491

[B30] de Azevedo Neto A. D. Prisco J. T. Enéas-Filho J. Rolim Medeiros J.-V. Gomes-Filho E. (2005). Hydrogen peroxide pre-treatment induces salt-stress acclimation in maize plants. J. Plant Physiol. 162, 1114–1122. doi: 10.1016/j.jplph.2005.01.007, PMID: 16255169

[B31] Ding H. He J. Wu Y. Wu X. Ge C. Wang Y.. (2018). The tomato mitogen-activated protein kinase slMPK1 is as a negative regulator of the high-temperature stress response. Plant Physiol. 177, 633–651. doi: 10.1104/pp.18.00067, PMID: 29678861 PMC6001329

[B32] Ding Y. Huang L. Jiang Q. Zhu C. (2020). MicroRNAs as important regulators of heat stress responses in plants. J. Agric. Food Chem. 68, 11320–11326. doi: 10.1021/acs.jafc.0c03597, PMID: 32870674

[B33] Doğru A. (2021). Effects of heat stress on photosystem II activity and antioxidant enzymes in two maize cultivars. Planta 253, 85. doi: 10.1007/s00425-021-03611-6, PMID: 33788056

[B34] Duc N. H. Csintalan Z. Posta K. (2018). Arbuscular mycorrhizal fungi mitigate negative effects of combined drought and heat stress on tomato plants. Plant Physiol. Biochem. 132, 297–307. doi: 10.1016/j.plaphy.2018.09.011, PMID: 30245343

[B35] Elakhdar A. Solanki S. Kubo T. Abed A. Elakhdar I. Khedr R.. (2022). Barley with improved drought tolerance: Challenges and perspectives. Environ. Exp. Bot. 201, 104965. doi: 10.1016/j.envexpbot.2022.104965

[B36] El-Maarouf-Bouteau H. Meimoun P. Job C. Job D. Bailly C. (2013). Role of protein and mRNA oxidation in seed dormancy and germination. Front. Plant Sci. 4. doi: 10.3389/fpls.2013.00077, PMID: 23579315 PMC3619121

[B37] El-Sappah A. H. Rather S. A. Wani S. H. Elrys A. S. Bilal M. Huang Q.. (2022). Heat stress-mediated constraints in maize (Zea mays) production: challenges and solutions. Front. Plant Sci. 13. doi: 10.3389/fpls.2022.879366, PMID: 35615131 PMC9125997

[B38] Falcioni R. Chicati M. L. de Oliveira R. B. Antunes W. C. Hasanuzzaman M. Demattê J. A. M.. (2024). Decreased Photosynthetic Efficiency in Nicotiana tabacum L. under Transient Heat Stress. Plants 13, 395. doi: 10.3390/plants13030395, PMID: 38337928 PMC10856914

[B39] FAO. (2021). World food and agriculture—statistical yearbook 2021. (Rome, Italy: FAO (Food and Agriculture Organization of the United Nations). Available online at: https://www.fao.org/documents/card/en/c/CB4477EN. (Accessed July 27, 2025).

[B40] Faralli M. Bontempo L. Bianchedi P. L. Moser C. Bertamini M. Lawson T.. (2022). Natural variation in stomatal dynamics drives divergence in heat stress tolerance and contributes to seasonal intrinsic water-use efficiency in Vitis vinifera (subsp. sativa and sylvestris). J. Exp. Bot. 73, 3238–3250. doi: 10.1093/jxb/erab552, PMID: 34929033

[B41] Farooq M. Aziz T. Basra S. M. A. Cheema M. A. Rehman H. (2008). Chilling tolerance in hybrid maize induced by seed priming with salicylic acid. J. Agron. Crop Sci. 194, 161–168. doi: 10.1111/j.1439-037X.2008.00300.x

[B42] Farooq A. Farooq N. Akbar H. Hassan Z. U. Gheewala S. H. (2023). A critical review of climate change impact at a global scale on cereal crop production. Agronomy 13, 162. doi: 10.3390/agronomy13010162

[B43] Feng D. Jia X. Yan Z. Li J. Gao J. Xiao W.. (2023). Underlying mechanisms of exogenous substances involved in alleviating plant heat stress. Plant Stress 10, 100288. doi: 10.1016/j.stress.2023.100288

[B44] Filippou P. Tanou G. Molassiotis A. Fotopoulos V. (2013). “Plant acclimation to environmental stress using priming agents,” in Plant Acclimation to Environmental Stress. Eds. Tuteja N. Singh Gill S. (Springer New York, New York, NY), 1–27. doi: 10.1007/978-1-4614-5001-6_1

[B45] Fortunato S. Lasorella C. Dipierro N. Vita F. de Pinto M. C. (2023). Redox signaling in plant heat stress response. Antioxidants 12, 605. doi: 10.3390/antiox12030605, PMID: 36978852 PMC10045013

[B46] Foyer C. H. Noctor G. (2013). Redox signaling in plants. Antioxidants Redox Signaling 18, 2087–2090. doi: 10.1089/ars.2013.5278, PMID: 23442120

[B47] Fu Y. Gu Q. Dong Q. Zhang Z. Lin C. Hu W.. (2019). Spermidine enhances heat tolerance of rice seeds by modulating endogenous starch and polyamine metabolism. Molecules 24, 1395. doi: 10.3390/molecules24071395, PMID: 30970602 PMC6480098

[B48] Gahlaut V. Baranwal V. K. Khurana P. (2018). miRNomes involved in imparting thermotolerance to crop plants. 3 Biotech. 8, 497. doi: 10.1007/s13205-018-1521-7, PMID: 30498670 PMC6261126

[B49] Gao Z. Su Y. Jiao G. Lou Z. Chang L. Yu R.. (2025). Cell-type specific miRNA regulatory network responses to ABA stress revealed by time series transcriptional atlases in Arabidopsis. Advanced Sci. 12, 2415083. doi: 10.1002/advs.202415083, PMID: 39792694 PMC11884551

[B50] Gill S. S. Tuteja N. (2010). Reactive oxygen species and antioxidant machinery in abiotic stress tolerance in crop plants. Plant Physiol. Biochem. 48, 909–930. doi: 10.1016/j.plaphy.2010.08.016, PMID: 20870416

[B51] Gondim F. A. Gomes-Filho E. Costa J. H. Mendes Alencar N. L. Prisco J. T. (2012). Catalase plays a key role in salt stress acclimation induced by hydrogen peroxide pretreatment in maize. Plant Physiol. Biochem. 56, 62–71. doi: 10.1016/j.plaphy.2012.04.012, PMID: 22609456

[B52] Greer D. H. Weedon M. M. (2012). Modelling photosynthetic responses to temperature of grapevine (Vitis vinifera cv. Semillon) leaves on vines grown in a hot climate. Plant Cell Environ. 35, 1050–1064. doi: 10.1111/j.1365-3040.2011.02471.x, PMID: 22150771

[B53] Guan Q. Lu X. Zeng H. Zhang Y. Zhu J. (2013). Heat stress induction of mi398 triggers a regulatory loop that is critical for thermotolerance in Arabidopsis. Plant J. 74, 840–851. doi: 10.1111/tpj.12169, PMID: 23480361

[B54] Gunderson C. A. Sholtis J. D. Wullschleger S. D. Tissue D. T. Hanson P. J. Norby R. J. (2002). Environmental and stomatal control of photosynthetic enhancement in the canopy of a sweetgum (Liquidambar styraciflua L.) plantation during 3 years of CO2 enrichment. Plant Cell Environ. 25, 379–393. doi: 10.1046/j.0016-8025.2001.00816.x

[B55] Guo T. Gull S. Ali M. M. Yousef A. F. Ercisli S. Kalaji H. M.. (2022). Heat stress mitigation in tomato (Solanum lycopersicum L.) through foliar application of gibberellic acid. Sci. Rep. 12, 11324. doi: 10.1038/s41598-022-15590-z, PMID: 35790780 PMC9256751

[B56] Halter G. Simonetti N. Suguitan C. Helm K. Soroksky J. Waters E. R. (2017). Patterns of thermotolerance, chlorophyll fluorescence, and heat shock gene expression vary among four Boechera species and Arabidopsis thaliana. Botany 95, 9–27. doi: 10.1139/cjb-2016-0158

[B57] Hamerlynck E. Knapp A. K. (1996). Photosynthetic and stomatal responses to high temperature and light in two oaks at the western limit of their range. Tree Physiol. 16, 557–565. doi: 10.1093/treephys/16.6.557, PMID: 14871709

[B58] Hasanuzzaman M. Hossain M. A. Fujita M. (2011). Nitric oxide modulates antioxidant defense and the methylglyoxal detoxification system and reduces salinity-induced damage of wheat seedlings. Plant Biotechnol. Rep. 5, 353–365. doi: 10.1007/s11816-011-0189-9 21264525

[B59] Hasanuzzaman M. Nahar K. Alam M. Roychowdhury R. Fujita M. (2013). Physiological, biochemical, and molecular mechanisms of heat stress tolerance in plants. Int. J. Mol. Sci. 14, 9643–9684. doi: 10.3390/ijms14059643, PMID: 23644891 PMC3676804

[B60] Hirayama T. Mochida K. (2022). Plant hormonomics: A key tool for deep physiological phenotyping to improve crop productivity. Plant Cell Physiol. 63, 1826–1839. doi: 10.1093/pcp/pcac067, PMID: 35583356 PMC9885943

[B61] Hönig M. Roeber V. M. Schmülling T. Cortleven A. (2023). Chemical priming of plant defense responses to pathogen attacks. Front. Plant Sci. 14. doi: 10.3389/fpls.2023.1146577, PMID: 37223806 PMC10200928

[B62] Huang Q. Zhang M. Li C. Li B. Zhuo S. Yang Y.. (2025). Response mechanism of water status and photosynthetic characteristics of Cotoneaster multiflorus under drought stress and rehydrated conditions. Front. Plant Sci. 15. doi: 10.3389/fpls.2024.1457955, PMID: 39877737 PMC11773621

[B63] Hussain I. Ashraf M. A. Rasheed R. Iqbal M. Ibrahim M. Ashraf S. (2016). Heat shock increases oxidative stress to modulate growth and physico-chemical attributes in diverse maize cultivars. Int. Agrophysics 30, 519–531. doi: 10.1515/intag-2016-0023

[B64] Hussain H. A. Men S. Hussain S. Chen Y. Ali S. Zhang S.. (2019a). Interactive effects of drought and heat stresses on morpho-physiological attributes, yield, nutrient uptake and oxidative status in maize hybrids. Sci. Rep. 9, 3890. doi: 10.1038/s41598-019-40362-7, PMID: 30846745 PMC6405865

[B65] Hussain I. Parveen A. Rasheed R. Ashraf M. A. Ibrahim M. Riaz S.. (2019b). Exogenous silicon modulates growth, physio-chemicals and antioxidants in barley (Hordeum vulgare L.) exposed to different temperature regimes. Silicon 11, 2753–2762. doi: 10.1007/s12633-019-0067-6

[B66] Ibañez C. Poeschl Y. Peterson T. Bellstädt J. Denk K. Gogol-Döring A.. (2017). Ambient temperature and genotype differentially affect developmental and phenotypic plasticity in Arabidopsis thaliana. BMC Plant Biol. 17, 114. doi: 10.1186/s12870-017-1068-5, PMID: 28683779 PMC5501000

[B67] Ihsan M. Z. Daur I. Alghabari F. Alzamanan S. Rizwan S. Ahmad M.. (2019). Heat stress and plant development: role of sulphur metabolites and management strategies. Acta Agriculturae Scandinavica Section B — Soil Plant Sci. 69, 332–342. doi: 10.1080/09064710.2019.1569715

[B68] IPCC (2023). “Summary for policymakers,” in Climate Change 2023: Synthesis Report. Contribution of Working Groups I, II, and III to the Sixth Assessment Report of the Intergovernmental Panel on Climate Change, eds. Lee H. Romero J. (Geneva, Switzerland: IPCC (Intergovernmental Panel on Climate Change), 1–34. Available online at: https://www.ipcc.ch/report/ar6/syr/downloads/report/IPCC_AR6_SYR_SPM.pdf. (Accessed July 26, 2025).

[B69] Iqbal M. Ashraf M. (2007). Seed preconditioning modulates growth, ionic relations, and photosynthetic capacity in adult plants of hexaploid wheat under salt stress. J. Plant Nutr. 30, 381–396. doi: 10.1080/01904160601171330

[B70] Iqbal S. Iqbal M. A. Li C. Iqbal A. Abbas R. N. (2023). Overviewing drought and heat stress amelioration—From plant responses to microbe-mediated mitigation. Sustainability 15, 1671. doi: 10.3390/su15021671

[B71] Ishibashi Y. Aoki N. Kasa S. Sakamoto M. Kai K. Tomokiyo R.. (2017). The interrelationship between abscisic acid and reactive oxygen species plays a key role in barley seed dormancy and germination. Front. Plant Sci. 8. doi: 10.3389/fpls.2017.00275, PMID: 28377774 PMC5359625

[B72] Ishibashi Y. Kasa S. Sakamoto M. Aoki N. Kai K. Yuasa T.. (2015). A role for reactive oxygen species produced by NADPH oxidases in the embryo and aleurone cells in barley seed germination. PloS One 10, e0143173. doi: 10.1371/journal.pone.0143173, PMID: 26579718 PMC4651353

[B73] Ishibashi Y. Yamaguchi H. Yuasa T. Iwaya-Inoue M. Arima S. Zheng S.-H. (2011). Hydrogen peroxide spraying alleviates drought stress in soybean plants. J. Plant Physiol. 168, 1562–1567. doi: 10.1016/j.jplph.2011.02.003, PMID: 21377755

[B74] Ito S. Hara T. Kawanami Y. Watanabe T. Thiraporn K. Ohtake N.. (2009). Carbon and nitrogen transport during grain filling in rice under high-temperature conditions. J. Agron. Crop Sci. 195, 368–376. doi: 10.1111/j.1439-037X.2009.00376.x

[B75] Jacott C. N. Boden S. A. (2020). Feeling the heat: developmental and molecular responses of wheat and barley to high ambient temperatures. J. Exp. Bot. 71, 5740–5751. doi: 10.1093/jxb/eraa326, PMID: 32667992 PMC7540836

[B76] Jagadish S. V. K. Murty M. V. R. Quick W. P. (2015). Rice responses to rising temperatures – challenges, perspectives and future directions. Plant Cell Environ. 38, 1686–1698. doi: 10.1111/pce.12430, PMID: 25142172

[B77] Jahan M. S. Md. M. Alotaibi F. S. Alabdallah N. M. Alharbi B. M. Ramadan K. M. A.. (2022). Exogenous putrescine increases heat tolerance in tomato seedlings by regulating chlorophyll metabolism and enhancing antioxidant defense efficiency. Plants 11, 1038. doi: 10.3390/plants11081038, PMID: 35448766 PMC9032913

[B78] Janni M. Maestri E. Gullì M. Marmiroli M. Marmiroli N. (2024). Plant responses to climate change, how global warming may impact on food security: a critical review. Front. Plant Sci. 14. doi: 10.3389/fpls.2023.1297569, PMID: 38250438 PMC10796516

[B79] Jardim-Messeder D. de Souza-Vieira Y. Sachetto-Martins G. (2025). Dressed up to the nines: the interplay of phytohormones signaling and redox metabolism during plant response to drought. Plants 14, 208. doi: 10.3390/plants14020208, PMID: 39861561 PMC11768152

[B80] Jiang L. Xiao M. Huang R. Wang J. (2025). The regulation of ROS and phytohormones in balancing crop yield and salt tolerance. Antioxidants 14, 63. doi: 10.3390/antiox14010063, PMID: 39857397 PMC11761564

[B81] Jin B. Wang L. Wang J. Jiang K.-Z. Wang Y. Jiang X.-X.. (2011). The effect of experimental warming on leaf functional traits, leaf structure and leaf biochemistry in Arabidopsis thaliana. BMC Plant Biol. 11, 35. doi: 10.1186/1471-2229-11-35, PMID: 21329528 PMC3045891

[B82] Kaczkowski B. Torarinsson E. Reiche K. Havgaard J. H. Stadler P. F. Gorodkin J. (2009). Structural profiles of human miRNA families from pairwise clustering. Bioinformatics 25, 291–294. doi: 10.1093/bioinformatics/btn628, PMID: 19059941

[B83] Kadyrbaev M. K. Golovatskaya I. F. Satkanov M. (2021). Features of regenerants morphogenesis and metabolism *in vitro*, obtained from different fragments of potato shoots. Vestnik Tomskogo Gosudarstvennogo Universiteta Biologiya 55, 114–134. doi: 10.17223/19988591/55/7

[B84] Khan M. A. Asaf S. Khan A. L. Jan R. Kang S.-M. Kim K.-M.. (2020). Extending thermotolerance to tomato seedlings by inoculation with SA1 isolate of Bacillus cereus and comparison with exogenous humic acid application. PloS One 15, e0232228. doi: 10.1371/journal.pone.0232228, PMID: 32353077 PMC7192560

[B85] Khan A. Khan V. Pandey K. Sopory S. K. Sanan-Mishra N. (2022). Thermo-priming mediated cellular networks for abiotic stress management in plants. Front. Plant Sci. 13. doi: 10.3389/fpls.2022.866409, PMID: 35646001 PMC9136941

[B86] Khan H. M. T. Mukhtar Balal R. Hussain Z. Ayyaz Javed S. Tauseef Jaffar M. Abdullah Alsahli A. (2024). Exogenous application of melatonin mitigate the heat stress in different tomato (Solanum lycopersicum L.) cultivars. J. King Saud Univ. - Sci. 36, 103086. doi: 10.1016/j.jksus.2023.103086

[B87] Khan A. L. Waqas M. Lee I.-J. (2015). Resilience of Penicillium resedanum LK6 and exogenous gibberellin in improving Capsicum annuum growth under abiotic stresses. J. Plant Res. 128, 259–268. doi: 10.1007/s10265-014-0688-1, PMID: 25537300

[B88] Khanna P. Kaur K. Gupta A. K. (2016). Salicylic acid induces differential antioxidant response in spring maize under high temperature stress. Indian J. Exp. Biol. 54, 386–393., PMID: 27468465

[B89] Kim J. J. Lee J. H. Kim W. Jung H. S. Huijser P. Ahn J. H. (2012). The microRNA156-SQUAMOSA PROMOTER BINDING PROTEIN-LIKE3 Module Regulates Ambient Temperature-Responsive Flowering via FLOWERING LOCUS T in Arabidopsis. Plant Physiol. 159, 461–478. doi: 10.1104/pp.111.192369, PMID: 22427344 PMC3375978

[B90] Klupczyńska E. A. Pawłowski T. A. (2021). Regulation of seed dormancy and germination mechanisms in a changing environment. Int. J. Mol. Sci. 22, 1357. doi: 10.3390/ijms22031357, PMID: 33572974 PMC7866424

[B91] Koevoets I. T. Venema J. H. Elzenga J. Theo. M. Testerink C. (2016). Roots withstanding their environment: exploiting root system architecture responses to abiotic stress to improve crop tolerance. Front. Plant Sci. 7. doi: 10.3389/fpls.2016.01335, PMID: 27630659 PMC5005332

[B92] Korkmaz A. Korkmaz Y. (2009). Promotion by 5-aminolevulenic acid of pepper seed germination and seedling emergence under low-temperature stress. Scientia Hortic. 119, 98–102. doi: 10.1016/j.scienta.2008.07.016

[B93] Kousar S. Ahmed F. Pervaiz A. Bojnec Š. (2021). Food insecurity, population growth, urbanization and water availability: the role of government stability. Sustainability 13, 12336. doi: 10.3390/su132212336

[B94] Kračun D. Lopes L. R. Cifuentes-Pagano E. Pagano P. J. (2025). NADPH oxidases: redox regulation of cell homeostasis and disease. Physiol. Rev. 105, 1291–1428. doi: 10.1152/physrev.00034.2023, PMID: 39814410 PMC12285607

[B95] Kruszka K. Pacak A. Swida-Barteczka A. Nuc P. Alaba S. Wroblewska Z.. (2014). Transcriptionally and post-transcriptionally regulated microRNAs in heat stress response in barley. J. Exp. Bot. 65, 6123–6135. doi: 10.1093/jxb/eru353, PMID: 25183744 PMC4203144

[B96] Krutovsky K. V. Popova A. A. Yakovlev I. A. Yanbaev Y. A. Matveev S. M. (2025). Response of pedunculate oak (Quercus robur L.) to adverse environmental conditions in genetic and dendrochronological studies. Plants 14, 109. doi: 10.3390/plants14010109, PMID: 39795368 PMC11723010

[B97] Kumar R. R. Rai G. K. Kota S. Watts A. Sakhare A. Kumar S.. (2023). Fascinating Dynamics of Silicon in alleviation of heat stress Induced oxidative damage in plants. Plant Growth Regul. 100, 321–335. doi: 10.1007/s10725-022-00879-w

[B98] Labouriau L. G. Osborn J. H. (1984). Temperature dependence of the germination of tomato seeds. J. Thermal Biol. 9, 285–294. doi: 10.1016/0306-4565(84)90010-X

[B99] Li X.-C. Chang C. Pei Z.-M. (2023). Reactive oxygen species in drought-induced stomatal closure: the potential roles of NPR1. Plants 12, 3194. doi: 10.3390/plants12183194, PMID: 37765358 PMC10537201

[B100] Li L. Chen G. Yuan M. Guo S. Wang Y. Sun J. (2022). CsbZIP2-miR9748-csNPF4.4 module mediates high temperature tolerance of cucumber through jasmonic acid pathway. Front. Plant Sci. 13. doi: 10.3389/fpls.2022.883876, PMID: 35574100 PMC9096661

[B101] Li Z.-G. Gong M. (2011). Mechanical stimulation-induced cross-adaptation in plants: an overview. J. Plant Biol. 54, 358–364. doi: 10.1007/s12374-011-9178-3

[B102] LI T. ZHANG X. LIU Q. LIU J. CHEN Y. SUI P. (2022). Yield penalty of maize (Zea mays L.) under heat stress in different growth stages: A review. J. Integr. Agric. 21, 2465–2476. doi: 10.1016/j.jia.2022.07.013

[B103] Li X. Zhuge S. Du J. Zhang P. Wang X. Liu T.. (2025). The molecular mechanism by which heat stress during the grain filling period inhibits maize grain filling and reduces yield. Front. Plant Sci. 15. doi: 10.3389/fpls.2024.1533527, PMID: 39898260 PMC11782181

[B104] Liang X. Wang D. Ye Q. Zhang J. Liu M. Liu H.. (2023). Stomatal responses of terrestrial plants to global change. Nat. Commun. 14, 2188. doi: 10.1038/s41467-023-37934-7, PMID: 37069185 PMC10110556

[B105] Lin J.-S. Kuo C.-C. Yang I.-C. Tsai W.-A. Shen Y.-H. Lin C.-C.. (2018). MicroRNA160 modulates plant development and heat shock protein gene expression to mediate heat tolerance in Arabidopsis. Front. Plant Sci. 9. doi: 10.3389/fpls.2018.00068, PMID: 29449855 PMC5799662

[B106] Liu J. Hasanuzzaman M. Wen H. Zhang J. Peng T. Sun H.. (2019). High temperature and drought stress cause abscisic acid and reactive oxygen species accumulation and suppress seed germination growth in rice. Protoplasma 256, 1217–1227. doi: 10.1007/s00709-019-01354-6, PMID: 31001689

[B107] Liu X.-M. Kim K. E. Kim K.-C. Nguyen X. C. Han H. J. Jung M. S.. (2010). Cadmium activates Arabidopsis MPK3 and MPK6 via accumulation of reactive oxygen species. Phytochemistry 71, 614–618. doi: 10.1016/j.phytochem.2010.01.005, PMID: 20116811

[B108] Liu X. Quan W. Bartels D. (2022). Stress memory responses and seed priming correlate with drought tolerance in plants: an overview. Planta 255, 45. doi: 10.1007/s00425-022-03828-z, PMID: 35066685 PMC8784359

[B109] Liu S.-J. Xu H.-H. Wang W.-Q. Li N. Wang W.-P. Møller I. M.. (2015). A proteomic analysis of rice seed germination as affected by high temperature and ABA treatment. Physiologia Plantarum 154, 142–161. doi: 10.1111/ppl.12292, PMID: 25270993

[B110] Louis N. Dhankher O. P. Puthur J. T. (2023). Seed priming can enhance and retain stress tolerance in ensuing generations by inducing epigenetic changes and trans-generational memory. Physiologia Plantarum 175, e13881. doi: 10.1111/ppl.13881, PMID: 36840678

[B111] Luo L. Cui Y. Ouyang N. Huang S. Gong X. Wei L.. (2025). Tolerance to multiple abiotic stresses is mediated by interacting CNGC proteins that regulate Ca2+ influx and stomatal movement in rice. J. Integr. Plant Biol. 67, 226–242. doi: 10.1111/jipb.13829, PMID: 39776199

[B112] Ma Z. Hu L. (2023). MicroRNA: A dynamic player from signalling to abiotic tolerance in plants. Int. J. Mol. Sci. 24, 11364. doi: 10.3390/ijms241411364, PMID: 37511124 PMC10379455

[B113] Ma J. Zhao P. Liu S. Yang Q. Guo H. (2020). The control of developmental phase transitions by microRNAs and their targets in seed plants. Int. J. Mol. Sci. 21, 1971. doi: 10.3390/ijms21061971, PMID: 32183075 PMC7139601

[B114] Macabuhay A. Arsova B. Watt M. Nagel K. A. Lenz H. Putz A.. (2022). Plant growth promotion and heat stress amelioration in Arabidopsis inoculated with paraburkholderia phytofirmans psJN rhizobacteria quantified with the growScreen-agar II phenotyping platform. Plants 11, 2927. doi: 10.3390/plants11212927, PMID: 36365381 PMC9655538

[B115] Mahalingam R. Duhan N. Kaundal R. Smertenko A. Nazarov T. Bregitzer P. (2022). Heat and drought induced transcriptomic changes in barley varieties with contrasting stress response phenotypes. Front. Plant Sci. 13. doi: 10.3389/fpls.2022.1066421, PMID: 36570886 PMC9772561

[B116] Mahmood A. Ali I. Wang W. Ata-Ul-Karim S. T. Liu B. Liu L.. (2022). Individual and combined effects of high-temperature stress at booting and flowering stages on rice grain yield. Agronomy 12, 3092. doi: 10.3390/agronomy12123092 39257042

[B117] Mahmood A. Wang W. Ali I. Zhen F. Osman R. Liu B.. (2021). Individual and combined effects of booting and flowering high-temperature stress on rice biomass accumulation. Plants 10, 1021. doi: 10.3390/plants10051021, PMID: 34065233 PMC8160744

[B118] Mallory A. C. Bartel D. P. Bartel B. (2005). MicroRNA-directed regulation of arabidopsis AUXIN RESPONSE FACTOR17 is essential for proper development and modulates expression of early auxin response genes. Plant Cell 17, 1360–1375. doi: 10.1105/tpc.105.031716, PMID: 15829600 PMC1091760

[B119] Marchin R. M. Backes D. Ossola A. Leishman M. R. Tjoelker M. G. Ellsworth D. S. (2022). Extreme heat increases stomatal conductance and drought-induced mortality risk in vulnerable plant species. Global Change Biol. 28, 1133–1146. doi: 10.1111/gcb.15976, PMID: 34741566 PMC9299030

[B120] Marchin R. M. Medlyn B. E. Tjoelker M. G. Ellsworth D. S. (2023). Decoupling between stomatal conductance and photosynthesis occurs under extreme heat in broadleaf tree species regardless of water access. Global Change Biol. 29, 6319–6335. doi: 10.1111/gcb.16929, PMID: 37698501

[B121] Mathur S. Agnihotri R. Sharma M. P. Reddy V. R. Jajoo A. (2021). Effect of high-temperature stress on plant physiological traits and mycorrhizal symbiosis in maize plants. J. Fungi 7, 867. doi: 10.3390/jof7100867, PMID: 34682289 PMC8539748

[B122] Mathur S. Agrawal D. Jajoo A. (2014). Photosynthesis: Response to high temperature stress. J. Photochem. Photobiol. B: Biol. 137, 116–126. doi: 10.1016/j.jphotobiol.2014.01.010, PMID: 24796250

[B123] May P. Liao W. Wu Y. Shuai B. Richard McCombie W. Zhang M. Q.. (2013). The effects of carbon dioxide and temperature on microRNA expression in Arabidopsis development. Nat. Commun. 4, 2145. doi: 10.1038/ncomms3145, PMID: 23900278

[B124] Medina E. Kim S.-H. Yun M. Choi W.-G. (2021). Recapitulation of the function and role of ROS generated in response to heat stress in plants. Plants 10, 371. doi: 10.3390/plants10020371, PMID: 33671904 PMC7918971

[B125] Mei Y. Song S. (2010). Response to temperature stress of reactive oxygen species scavenging enzymes in the cross-tolerance of barley seed germination. J. Zhejiang Univ. Sci. B 11, 965–972. doi: 10.1631/jzus.B1000147, PMID: 21121076 PMC2997406

[B126] Miao R. Liu X. Zhao Y. Zhao Y. Dong H. Huang G.. (2025). Beneficial roles of 1-MCP on regulation of photosynthetic electron transport and energy dissipation in chrysanthemum under heat stress. Horticulturae 11, 68. doi: 10.3390/horticulturae11010068

[B127] Mirón I. J. Linares C. Díaz J. (2023). The influence of climate change on food production and food safety. Environ. Res. 216, 114674. doi: 10.1016/j.envres.2022.114674, PMID: 36341795

[B128] Mostofa M. G. Fujita M. (2013). Salicylic acid alleviates copper toxicity in rice (Oryza sativa L.) seedlings by up-regulating antioxidative and glyoxalase systems. Ecotoxicology 22, 959–973. doi: 10.1007/s10646-013-1073-x, PMID: 23579392

[B129] Mostofa M. G. Seraj Z. I. Fujita M. (2014). Exogenous sodium nitroprusside and glutathione alleviate copper toxicity by reducing copper uptake and oxidative damage in rice (Oryza sativa L.) seedlings. Protoplasma 251, 1373–1386. doi: 10.1007/s00709-014-0639-7, PMID: 24752795

[B130] Msarie M. W. Methela N. J. Islam M. S. An T. H. Das A. K. Lee D.-S.. (2025). Enhancing soybean salt tolerance with GSNO and silicon: A comprehensive physiological, biochemical, and genetic study. Int. J. Mol. Sci. 26, 609. doi: 10.3390/ijms26020609, PMID: 39859323 PMC11765656

[B131] Mukhtar T. Ali F. Rafique M. Ali J. Afridi M. S. Smith D.. (2023). Biochemical Characterization and Potential of Bacillus safensis Strain SCAL1 to Mitigate Heat Stress in Solanum lycopersicum L. J. Plant Growth Regul. 42, 523–538. doi: 10.1007/s00344-021-10571-4

[B132] Mukhtar T. Rehman S. U. Smith D. Sultan T. Seleiman M. F. Alsadon A. A.. (2020). Mitigation of Heat Stress in Solanum lycopersicum L. by ACC-deaminase and Exopolysaccharide Producing Bacillus cereus: Effects on Biochemical Profiling. Sustainability 12, 2159. doi: 10.3390/su12062159

[B133] Murad Lima A. C. Brichi L. Trevisan L. R. Leão de Souza Dominguez A. Nocera Santiago G. Gomes T. M.. (2025). Effects of irrigation with treated slaughterhouse effluent and Bradyrhizobium spp. Inoculation on soybean development and productivity: strategies for sustainable management. Agronomy 15, 167. doi: 10.3390/agronomy15010167

[B134] Nakajima M. Yamaguchi I. Kizawa S. Murofushi N. Takahashi N. (1991). Semi-quantification of GAX and GA4 in male-sterile anthers of rice by radioimmunoassay. Plant Cell Physiol. 32, 511–513. doi: 10.1093/oxfordjournals.pcp.a078109

[B135] Narendra Babu A. Jogaiah S. Ito S. Kestur Nagaraj A. Tran L.-S. P. (2015). Improvement of growth, fruit weight and early blight disease protection of tomato plants by rhizosphere bacteria is correlated with their beneficial traits and induced biosynthesis of antioxidant peroxidase and polyphenol oxidase. Plant Sci. 231, 62–73. doi: 10.1016/j.plantsci.2014.11.006, PMID: 25575992

[B136] Naz R. Gul F. Zahoor S. Nosheen A. Yasmin H. Keyani R.. (2022). Interactive effects of hydrogen sulphide and silicon enhance drought and heat tolerance by modulating hormones, antioxidant defence enzymes and redox status in barley (Hordeum vulgare L.). Plant Biol. 24, 684–696. doi: 10.1111/plb.13374, PMID: 34879172

[B137] Noctor G. Reichheld J.-P. Foyer C. H. (2018). ROS-related redox regulation and signaling in plants. Semin. Cell Dev. Biol. 80, 3–12. doi: 10.1016/j.semcdb.2017.07.013, PMID: 28733165

[B138] Nurbekova Z. Satkanov M. Beisekova M. Akbassova A. Ualiyeva R. Cui J.. (2024). Strategies for achieving high and sustainable plant productivity in saline soil conditions. Horticulturae 10, 878. doi: 10.3390/horticulturae10080878

[B139] Ostendarp M. de Breuyn M. El-Khaled Y. C. Garcias-Bonet N. Carvalho S. Peixoto R. S.. (2025). Temperature-dependent responses of the hard corals Acropora sp. and Pocillopora verrucosa to molecular hydrogen. PloS One 20, e0308894. doi: 10.1371/journal.pone.0308894, PMID: 40132032 PMC11936180

[B140] Pagano A. Macovei A. Balestrazzi A. (2023). Molecular dynamics of seed priming at the crossroads between basic and applied research. Plant Cell Rep. 42, 657–688. doi: 10.1007/s00299-023-02988-w, PMID: 36780009 PMC9924218

[B141] Pérez-Bueno M. L. Illescas-Miranda J. Martín-Forero A. F. de Marcos A. Barón M. Fenoll C.. (2022). An extremely low stomatal density mutant overcomes cooling limitations at supra-optimal temperature by adjusting stomatal size and leaf thickness. Front. Plant Sci. 13. doi: 10.3389/fpls.2022.919299, PMID: 35937324 PMC9355609

[B142] Pieterse C. M. Zamioudis C. Berendsen R. L. Weller D. M. Van Wees S. C. Bakker P. A. (2014). Induced systemic resistance by beneficial microbes. Annu. Rev. Phytopathol. 52, 347–375. doi: 10.1146/annurev-phyto-082712-102340, PMID: 24906124

[B143] Qaderi M. M. (2023). Environmental regulation of weed seed dormancy and germination. Seeds 2, 259–277. doi: 10.3390/seeds2030020

[B144] Qin K. Ye X. Luo S. Fernie A. R. Zhang Y. (2025). Engineering carbon assimilation in plants. J. Integr. Plant Biol. 67, 926–948. doi: 10.1111/jipb.13825, PMID: 39783795

[B145] Qu Y. Mueller-Cajar O. Yamori W. (2023). Improving plant heat tolerance through modification of Rubisco activase in C3 plants to secure crop yield and food security in a future warming world. J. Exp. Bot. 74, 591–599. doi: 10.1093/jxb/erac340, PMID: 35981868

[B146] Raghunath M. Beena R. Mohan V. Viji M. Manju R. Stephen R. (2021). High temperature stress mitigation in rice (Oryza sativa L.): Foliar application of plant growth regulators and nutrients. J. Crop Weed 17, 34–47. doi: 10.22271/09746315.2021.v17.i1.1404

[B147] Rajametov S. N. Yang E. Y. Cho M. C. Chae S. Y. Jeong H. B. Chae W. B. (2021). Heat-tolerant hot pepper exhibits constant photosynthesis via increased transpiration rate, high proline content and fast recovery in heat stress condition. Sci. Rep. 11, 14328. doi: 10.1038/s41598-021-93697-5, PMID: 34253784 PMC8275607

[B148] Rao M. J. Zheng B. (2025). The role of polyphenols in abiotic stress tolerance and their antioxidant properties to scavenge reactive oxygen species and free radicals. Antioxidants 14, 74. doi: 10.3390/antiox14010074, PMID: 39857408 PMC11761259

[B149] Raza S. H. A. Abdelnour S. A. Dhshan A. I. M. Hassanin A. A. Noreldin A. E. Albadrani G. M.. (2021). Potential role of specific microRNAs in the regulation of thermal stress response in livestock. J. Thermal Biol. 96, 102859. doi: 10.1016/j.jtherbio.2021.102859, PMID: 33627286

[B150] Redman R. S. Kim Y. O. Woodward C. J. D. A. Greer C. Espino L. Doty S. L.. (2011). Increased fitness of rice plants to abiotic stress via habitat adapted symbiosis: A strategy for mitigating impacts of climate change. PloS One 6, e14823. doi: 10.1371/journal.pone.0014823, PMID: 21750695 PMC3130040

[B151] Reinhart B. J. Weinstein E. G. Rhoades M. W. Bartel B. Bartel D. P. (2002). MicroRNAs in plants. Genes Dev. 16, 1616–1626. doi: 10.1101/gad.1004402, PMID: 12101121 PMC186362

[B152] Ren H. Bao J. Gao Z. Sun D. Zheng S. Bai J. (2023). How rice adapts to high temperatures. Front. Plant Sci. 14. doi: 10.3389/fpls.2023.1137923, PMID: 37008476 PMC10063981

[B153] Rollins J. A. Habte E. Templer S. E. Colby T. Schmidt J. von Korff M. (2013). Leaf proteome alterations in the context of physiological and morphological responses to drought and heat stress in barley (Hordeum vulgare L.). J. Exp. Bot. 64, 3201–3212. doi: 10.1093/jxb/ert158, PMID: 23918963 PMC3733145

[B154] Ru C. Hu X. Chen D. Wang W. (2023). Droughts and thermo-priming enhance acclimation to later drought and heat stress in maize seedlings by improving leaf physiological activity. Agronomy 13, 1124. doi: 10.3390/agronomy13041124

[B155] Ruan M. Zhao H. Wen Y. Chen H. He F. Hou X.. (2024). The complex transcriptional regulation of heat stress response in maize. Stress Biol. 4, 24. doi: 10.1007/s44154-024-00165-x, PMID: 38668992 PMC11052759

[B156] Sailaja B. Anjum N. Vishnu Prasanth V. Sarla N. Subrahmanyam D. Voleti S. R.. (2014). Comparative study of susceptible and tolerant genotype reveals efficient recovery and root system contributes to heat stress tolerance in rice. Plant Mol. Biol. Rep. 32, 1228–1240. doi: 10.1007/s11105-014-0728-y

[B157] Samat A. Zhanassova K. Soltabayeva A. Syzdyk K. Akbassova A. Zhangazin S.. (2024). The role of small RNAs under abiotic stress in plants. BULLETIN of L.N. Gumilyov Eurasian National University 149, 50–62. doi: 10.32523/2616-7034-2024-149-4-50-62

[B158] Sarma B. Kashtoh H. Lama Tamang T. Bhattacharyya P. N. Mohanta Y. K. Baek K.-H. (2023). Abiotic stress in rice: visiting the physiological response and its tolerance mechanisms. Plants 12, 3948. doi: 10.3390/plants12233948, PMID: 38068585 PMC10708142

[B159] Sathiyaraj G. Srinivasan S. Kim Y.-J. Lee O. R. Parvin S. Balusamy S. R. D.. (2014). Acclimation of hydrogen peroxide enhances salt tolerance by activating defense-related proteins in Panax ginseng C.A. Meyer. Mol. Biol. Rep. 41, 3761–3771. doi: 10.1007/s11033-014-3241-3, PMID: 24584574

[B160] Scafaro A. P. Posch B. C. Evans J. R. Farquhar G. D. Atkin O. K. (2023). Rubisco deactivation and chloroplast electron transport rates co-limit photosynthesis above optimal leaf temperature in terrestrial plants. Nat. Commun. 14, 2820. doi: 10.1038/s41467-023-38496-4, PMID: 37198175 PMC10192301

[B161] Scandalios J. G. Acevedo A. Ruzsa S. (2000). Catalase gene expression in response to chronic high temperature stress in maize. Plant Sci. 156, 103–110. doi: 10.1016/S0168-9452(00)00235-1, PMID: 10908810

[B162] Schoffl F. Prandl R. Reindl A. (1999). “Molecular responses to heat stress,” in Molecular responses to cold, drought, heat and salt stress in higher plants, eds. Shinozaki K. Yamaguchi-Shinozaki K. (Austin, TX: R.G. Landes Co.), 81–98.

[B163] Secomandi E. De Gregorio M. A. Castro-Cegrí A. Lucini L. (2025). Biochemical, photosynthetic and metabolomics insights of single and combined effects of salinity, heat, cold and drought in Arabidopsis. Physiologia Plantarum 177, e70062. doi: 10.1111/ppl.70062, PMID: 39821073 PMC11739553

[B164] Shimizu M. Kuno K. (1967). Some cyto-histological observations on the morphogenetically abnormal rice spikelets caused by a low temperature. Japanese J. Crop Sci. 36, 489–502. doi: 10.1626/JCS.36.4_489

[B165] Singh A. K. Mishra P. Kashyap S. P. Karkute S. G. Singh P. M. Rai N.. (2022). Molecular insights into mechanisms underlying thermo-tolerance in tomato. Front. Plant Sci. 13. doi: 10.3389/fpls.2022.1040532, PMID: 36388532 PMC9645296

[B166] Slot M. Garcia M. N. Winter K. (2016). Temperature response of CO2 exchange in three tropical tree species. Funct. Plant Biol. 43, 468–478. doi: 10.1071/FP15320, PMID: 32480477

[B167] Spychała J. Noweiska A. Tomkowiak A. Bobrowska R. Szewczyk K. Kwiatek M. T. (2025). Unraveling Effects of miRNAs Associated with APR Leaf Rust Resistance Genes in Hybrid Forms of Common Wheat (Triticum aestivum L.). Int. J. Mol. Sci. 26, 665. doi: 10.3390/ijms26020665, PMID: 39859380 PMC11766205

[B168] Ssemugenze B. Ocwa A. Kuunya R. Gumisiriya C. Bojtor C. Nagy J.. (2025). Enhancing maize production through timely nutrient supply: the role of foliar fertiliser application. Agronomy 15, 176. doi: 10.3390/agronomy15010176

[B169] Stief A. Altmann S. Hoffmann K. Pant B. D. Scheible W.-R. Bäurle I. (2014). Arabidopsis miR156 Regulates Tolerance to Recurring Environmental Stress through SPL Transcription Factors. Plant Cell 26, 1792–1807. doi: 10.1105/tpc.114.123851, PMID: 24769482 PMC4036586

[B170] Sunkar R. Kapoor A. Zhu J.-K. (2006). Posttranscriptional induction of two Cu/Zn superoxide dismutase genes in Arabidopsis is mediated by downregulation of miR398 and important for oxidative stress tolerance. Plant Cell 18, 2051–2065. doi: 10.1105/tpc.106.041673, PMID: 16861386 PMC1533975

[B171] Sunkar R. Zhu J.-K. (2004). Novel and stress-regulated microRNAs and other small RNAs from Arabidopsis. Plant Cell 16, 2001–2019. doi: 10.1105/tpc.104.022830, PMID: 15258262 PMC519194

[B172] Swain R. Rout G. R. (2017). “Silicon in agriculture,” in Sustainable Agriculture Reviews. Ed. Lichtfouse E. (Springer International Publishing, Cham), 233–260. doi: 10.1007/978-3-319-58679-3_8

[B173] Tang R. Mei C. Zhang J. Cai X. Wu G. (1996). Relationship between rice male sterility induction by TO_(3) and level of endogenous hormones. Jiangsu J. Agric. Sci. 12, 6–10.

[B174] Taratima W. Chuanchumkan C. Maneerattanarungroj P. Trunjaruen A. Theerakulpisut P. Dongsansuk A. (2022). Effect of Heat Stress on Some Physiological and Anatomical Characteristics of Rice (Oryza sativa L.) cv. KDML105 Callus and Seedling. Biology 11, 1587. doi: 10.3390/biology11111587, PMID: 36358287 PMC9687333

[B175] Teiba I. I. El-Bilawy E. H. Elsheery N. I. Rastogi A. (2024). Microbial allies in agriculture: harnessing plant growth-promoting microorganisms as guardians against biotic and abiotic stresses. Horticulturae 10, 12. doi: 10.3390/horticulturae10010012

[B176] Teng K. Li J. Liu L. Han Y. Du Y. Zhang J.. (2014). Exogenous ABA induces drought tolerance in upland rice: the role of chloroplast and ABA biosynthesis-related gene expression on photosystem II during PEG stress. Acta Physiologiae Plantarum 36, 2219–2227. doi: 10.1007/s11738-014-1599-4

[B177] Terán F. Vives-Peris V. Gómez-Cadenas A. Pérez-Clemente R. M. (2024). Facing climate change: plant stress mitigation strategies in agriculture. Physiologia Plantarum 176, e14484. doi: 10.1111/ppl.14484, PMID: 39157905

[B178] Tokić M. Leljak Levanić D. Ludwig-Müller J. Bauer N. (2023). Growth and molecular responses of tomato to prolonged and short-term heat exposure. Int. J. Mol. Sci. 24, 4456. doi: 10.3390/ijms24054456, PMID: 36901887 PMC10002527

[B179] Tran B.-L. Tseng W.-C. Chen C.-C. (2025). Climate change impacts on crop yields across temperature rise thresholds and climate zones. Sci. Rep. 15, 23424. doi: 10.1038/s41598-025-07405-8, PMID: 40603482 PMC12223106

[B180] Veloo K. Zúñiga Espinoza C. Salgado A. E. Jacoby P. W. Sankaran S. (2025). Multispectral, thermal, and hyperspectral sensing data depict stomatal conductance in grapevine. Remote Sens. 17, 137. doi: 10.3390/rs17010137

[B181] Vile D. Pervent M. Belluau M. Vasseur F. Bresson J. Muller B.. (2012). Arabidopsis growth under prolonged high temperature and water deficit: independent or interactive effects? Plant Cell Environ. 35, 702–718. doi: 10.1111/j.1365-3040.2011.02445.x, PMID: 21988660

[B182] Visakorpi K. Block S. Pellissier L. Levine J. M. Alexander J. (2023). Eco-physiological and morphological traits explain alpine plant species’ response to warming. Funct. Ecol. 37, 287–301. doi: 10.3929/ethz-b-000587865

[B183] Voinnet O. (2009). Origin, biogenesis, and activity of plant microRNAs. Cell 136, 669–687. doi: 10.1016/j.cell.2009.01.046, PMID: 19239888

[B184] Wahid A. Sehar S. Perveen M. Gelani S. Basra S. Farooq M. (2008). Seed pretreatment with hydrogen peroxide improves heat tolerance in maize at germination and seedling growth stages. Seed Sci. Technol. 36, 633–645. doi: 10.15258/sst.2008.36.3.13

[B185] Walter J. Jentsch A. Beierkuhnlein C. Kreyling J. (2013). Ecological stress memory and cross stress tolerance in plants in the face of climate extremes. Environ. Exp. Bot. 94, 3–8. doi: 10.1016/j.envexpbot.2012.02.009

[B186] Wang L.-J. Fan L. Loescher W. Duan W. Liu G.-J. Cheng J.-S.. (2010a). Salicylic acid alleviates decreases in photosynthesis under heat stress and accelerates recovery in grapevine leaves. BMC Plant Biol. 10, 34. doi: 10.1186/1471-2229-10-34, PMID: 20178597 PMC2848757

[B187] Wang Y. Li J. Wang J. Li Z. (2010b). Exogenous H2O2 improves the chilling tolerance of manilagrass and mascarenegrass by activating the antioxidative system. Plant Growth Regul. 61, 195–204. doi: 10.1007/s10725-010-9470-0

[B188] Wang L. Ma K.-B. Lu Z.-G. Ren S.-X. Jiang H.-R. Cui J.-W.. (2020). Differential physiological, transcriptomic and metabolomic responses of Arabidopsis leaves under prolonged warming and heat shock. BMC Plant Biol. 20, 86. doi: 10.1186/s12870-020-2292-y, PMID: 32087683 PMC7036190

[B189] Waqas M. Khan A. L. Shahzad R. Ullah I. Khan A. R. Lee I.-J. (2015). Mutualistic fungal endophytes produce phytohormones and organic acids that promote japonica rice plant growth under prolonged heat stress. J. Zhejiang University-SCIENCE B 16, 1011–1018. doi: 10.1631/jzus.B1500081, PMID: 26642184 PMC4686363

[B190] Wijewardene I. Shen G. Zhang H. (2021). Enhancing crop yield by using Rubisco activase to improve photosynthesis under elevated temperatures. Stress Biol. 1, 2. doi: 10.1007/s44154-021-00002-5, PMID: 37676541 PMC10429496

[B191] WMO (2024). WMO Global Annual to Decadal Climate Update: Target years: 2024 and 2024–2028. (Geneva, Switzerland: WMO (World Meteorological Organization)). Available online at: https://library.wmo.int/idurl/4/68910. (Accessed July 26, 2025).

[B192] Wu G. Park M. Y. Conway S. R. Wang J.-W. Weigel D. Poethig R. S. (2009). The Sequential Action of miR156 and miR172 Regulates Developmental Timing in Arabidopsis. Cell 138, 750–759. doi: 10.1016/j.cell.2009.06.031, PMID: 19703400 PMC2732587

[B193] Wu C. Tang S. Li G. Wang S. Fahad S. Ding Y. (2019). Roles of phytohormone changes in the grain yield of rice plants exposed to heat: a review. PeerJ 7, e7792. doi: 10.7717/peerj.7792, PMID: 31763066 PMC6873875

[B194] Wu T. Tissue D. T. Jiang M. Slot M. Crous K. Y. Yuan J.. (2025). Leaf photosynthetic and respiratory thermal acclimation in terrestrial plants in response to warming: A global synthesis. Global Change Biol. 31, e70026. doi: 10.1111/gcb.70026, PMID: 39825386

[B195] Xia Z. Zhang G. Zhang S. Wang Q. Fu Y. Lu H. (2021). Efficacy of root zone temperature increase in root and shoot development and hormone changes in different maize genotypes. Agriculture 11, 477. doi: 10.3390/agriculture11060477

[B196] Yamaura H. Kanno K. Takano N. Isozaki M. Iwasaki Y. (2021). Supra-optimal daily mean temperature stimulates plant growth and carbohydrate use in tomato. Scientia Hortic. 276, 109780. doi: 10.1016/j.scienta.2020.109780

[B197] Yang J. Chen X. Zhu C. Peng X. He X. Fu J.. (2018). Effects of high temperature on yield, quality and physiological components of early rice. Pak. J. Agric. Sci. 55, 13–22. doi: 10.21162/PAKJAS/18.2621

[B198] Yang X. Dong G. Palaniappan K. Mi G. Baskin T. I. (2017). Temperature-compensated cell production rate and elongation zone length in the root of Arabidopsis thaliana. Plant Cell Environ. 40, 264–276. doi: 10.1111/pce.12855, PMID: 27813107

[B199] Yang Z. Jiang Y. Qiu R. Gong X. Agathokleous E. Hu W.. (2023). Heat stress decreased transpiration but increased evapotranspiration in gerbera. Front. Plant Sci. 14, 1119076. doi: 10.3389/fpls.2023.1119076, PMID: 36743492 PMC9892838

[B200] Yang Z. Man J. Liu H. Wu D. Gu Q. Zhang H.. (2025). Study on the *in vitro* and *in vivo* antioxidant activity and potential mechanism of polygonum viviparum L. Antioxidants 14, 41. doi: 10.3390/antiox14010041, PMID: 39857375 PMC11762547

[B201] Yang D. Peng S. Wang F. (2020). Response of photosynthesis to high growth temperature was not related to leaf anatomy plasticity in rice (Oryza sativa L.). Front. Plant Sci. 11. doi: 10.3389/fpls.2020.00026, PMID: 32117372 PMC7018767

[B202] Zahra N. Hafeez M. B. Ghaffar A. Kausar A. Zeidi M. A. Siddique K. H. M.. (2023). Plant photosynthesis under heat stress: Effects and management. Environ. Exp. Bot. 206, 105178. doi: 10.1016/j.envexpbot.2022.105178

[B203] Zahra M. Rasul S. Saeed F. Rasool L. Manzoor H. (2022). Exogenous Application of Salicylic acid Mitigates heat-induced Oxidative Stress in Barley (Hordeum vulgare L.). PJBB 3, 133–143. doi: 10.52700/pjbb.v3i2.177

[B204] Zeng J. Dong Z. Wu H. Tian Z. Zhao Z. (2017). Redox regulation of plant stem cell fate. EMBO J. 36, 2844–2855. doi: 10.15252/embj.201695955, PMID: 28838936 PMC5623875

[B205] Zhanassova K. Kurmanbayeva A. Gadilgereyeva B. Yermukhambetova R. Iksat N. Amanbayeva U.. (2021). ROS status and antioxidant enzyme activities in response to combined temperature and drought stresses in barley. Acta Physiologiae Plantarum 43, 114. doi: 10.1007/s11738-021-03281-7

[B206] Zhang M. An P. Li H. Wang X. Zhou J. Dong P.. (2019). The miRNA-mediated post-transcriptional regulation of maize in response to high temperature. Int. J. Mol. Sci. 20, 1754. doi: 10.3390/ijms20071754, PMID: 30970661 PMC6480492

[B207] Zhang X. Rademacher T. Liu H. Wang L. Manzanedo R. D. (2023). Fading regulation of diurnal temperature ranges on drought-induced growth loss for drought-tolerant tree species. Nat. Commun. 14, 6916. doi: 10.1038/s41467-023-42654-z, PMID: 37903773 PMC10616191

[B208] Zhang Q. L. Wei Y. X. Peng C. L. (2018). Effects of endogenous ascorbic acid on resistance to high-temperature stress in excised rice leaves. Photosynthetica 56, 1453–1458. doi: 10.1007/s11099-018-0836-2

[B209] Zhang F. Yang J. Zhang N. Wu J. Si H. (2022). Roles of microRNAs in abiotic stress response and characteristics regulation of plant. Front. Plant Sci. 13. doi: 10.3389/fpls.2022.919243, PMID: 36092392 PMC9459240

[B210] Zhao J. He Q. Chen G. Wang L. Jin B. (2016). Regulation of non-coding RNAs in heat stress responses of plants. Front. Plant Sci. 7. doi: 10.3389/fpls.2016.01213, PMID: 27588021 PMC4988968

[B211] Zhao C. Liu B. Piao S. Wang X. Lobell D. B. Huang Y.. (2017). Temperature increase reduces global yields of major crops in four independent estimates. Proc. Natl. Acad. Sci. 114, 9326–9331. doi: 10.1073/pnas.1701762114, PMID: 28811375 PMC5584412

[B212] Zhao Q. Zhou L. Liu J. Du X. Asad M.-A.-U. Huang F.. (2018). Relationship of ROS accumulation and superoxide dismutase isozymes in developing anther with floret fertility of rice under heat stress. Plant Physiol. Biochem. 122, 90–101. doi: 10.1016/j.plaphy.2017.11.009, PMID: 29202329

[B213] Zheng Y. Cai Z. Wang Z. Maruza T. M. Zhang G. (2025). The genetics and breeding of heat stress tolerance in wheat: advances and prospects. Plants 14, 148. doi: 10.3390/plants14020148, PMID: 39861500 PMC11768744

[B214] Zhou R. Kjær K. H. Rosenqvist E. Yu X. Wu Z. Ottosen C.-O. (2017). Physiological response to heat stress during seedling and anthesis stage in tomato genotypes differing in heat tolerance. J. Agron. Crop Sci. 203, 68–80. doi: 10.1111/jac.12166

[B215] Zhou R. Kong L. Wu Z. Rosenqvist E. Wang Y. Zhao L.. (2019). Physiological response of tomatoes at drought, heat and their combination followed by recovery. Physiologia Plantarum 165, 144–154. doi: 10.1111/ppl.12764, PMID: 29774556

[B216] Zhou M. Wu Y. Yang Y. Yuan Y. Lin J. Lin L.. (2025). Trade-off between enzymatic antioxidant defense and accumulation of organic metabolite affects salt tolerance of white clover associated with redox, water, and metabolic homeostases. Plants 14, 145. doi: 10.3390/plants14020145, PMID: 39861499 PMC11768267

[B217] Zhuo J. Wang W. Lu Y. Sen W. Wang X. (2009). Osmopriming-regulated changes of plasma membrane composition and function were inhibited by phenylarsine oxide in soybean seeds. J. Integr. Plant Biol. 51, 858–867. doi: 10.1111/j.1744-7909.2009.00861.x, PMID: 19723245

[B218] Zolkiewicz K. Gruszka D. (2025). Take a deep BReath: Manipulating brassinosteroid homeostasis helps cereals adapt to environmental stress. Plant Physiol. 197, kiaf003. doi: 10.1093/plphys/kiaf003, PMID: 39761526 PMC11781206

